# Functional Characterization of a Global Virulence Regulator Hfq and Identification of Hfq-Dependent sRNAs in the Plant Pathogen *Pantoea ananatis*

**DOI:** 10.3389/fmicb.2019.02075

**Published:** 2019-09-11

**Authors:** Gi Yoon Shin, Jeffrey K. Schachterle, Divine Y. Shyntum, Lucy N. Moleleki, Teresa A. Coutinho, George W. Sundin

**Affiliations:** ^1^Centre for Microbial Ecology and Genomics, Forestry and Agricultural Biotechnology Institute, Department of Biochemistry, Genetics and Microbiology, University of Pretoria, Pretoria, South Africa; ^2^Forestry and Agricultural Biotechnology Institute, Department of Biochemistry, Genetics and Microbiology, University of Pretoria, Pretoria, South Africa; ^3^Department of Plant, Soil and Microbial Sciences, College of Agriculture & Natural Resources, Michigan State University, East Lansing, MI, United States

**Keywords:** *Pantoea ananatis*, plant pathogen, Hfq, sRNA, regulation, virulence

## Abstract

To successfully infect plant hosts, the collective regulation of virulence factors in a bacterial pathogen is crucial. Hfq is an RNA chaperone protein that facilitates the small RNA (sRNA) regulation of global gene expression at the post-transcriptional level. In this study, the functional role of Hfq in a broad host range phytopathogen *Pantoea ananatis* was determined. Inactivation of the *hfq* gene in *P. ananatis* LMG 2665^T^ resulted in the loss of pathogenicity and motility. In addition, there was a significant reduction of quorum sensing signal molecule acyl-homoserine lactone (AHL) production and biofilm formation. Differential sRNA expression analysis between the *hfq* mutant and wild-type strains of *P. ananatis* revealed 276 sRNAs affected in their abundance by the loss of *hfq* at low (OD_600_ = 0.2) and high cell (OD_600_ = 0.6) densities. Further analysis identified 25 Hfq-dependent sRNAs, all showing a predicted Rho-independent terminator of transcription and mapping within intergenic regions of the *P. ananatis* genome. These included known sRNAs such as ArcZ, FnrS, GlmZ, RprA, RyeB, RyhB, RyhB2, Spot42, and SsrA, and 16 novel *P. ananatis* sRNAs. The current study demonstrated that Hfq is an important component of the collective regulation of virulence factors and sets a foundation for understanding Hfq-sRNA mediated regulation in the phytopathogen *P. ananatis*.

## Introduction

*Pantoea ananatis*, formerly described as the pineapple pathogen *Erwinia ananas* (Serrano, 1928), is a Gram-negative bacterium belonging to the family *Enterobacteriaceae*. To date, the occurrence of *P. ananatis* has been reported from various ecological niches spanning both the aquatic and terrestrial environments, including fresh (Morohoshi et al., 2007) and marine water (Jatt et al., 2014) as well as the rhizosphere of crop plants (Oliveira et al., 2008; Marquez-Santacruz et al., 2010). The bacterium exhibits ecologically diverse roles in association with its environment. For example, *P. ananatis* can be found as an epiphyte of crop and weed plants (Gitaitis et al., 2002) or as an endophyte in maize kernels (Rijavec et al., 2007) and rice seeds (Okunishi et al., 2005). Moreover, the ability of *P. ananatis* to solubilize phosphate, and produce indole-acetic acid and siderophores, makes the bacterium an ideal plant growth-promoting agent in the production of pepper (Kang et al., 2007), soybean (Kuklinsky-Sobral et al., 2004), and sugarcane (da Silva et al., 2015).

*Pantoea ananatis* is better known as a phytopathogen affecting the yield of many economically important plant species that causes blight and dieback of *Eucalyptus* (Coutinho et al., 2002), maize leaf spot disease and brown stalk rot (Goszczynska et al., 2006; Pérez-y-Terrón et al., 2009; Alippi and López, 2010; Krawczyk et al., 2010), leaf blight and bulb rot of onion (Gitaitis and Gay, 1997; Schwartz and Otto, 2000; Goszczynska et al., 2007), palea browning and stem necrosis of rice (Azegami et al., 1983; Cother et al., 2004; Cortesi and Pizzatti, 2007), and fruit rot of netted melon (Kido et al., 2008). *P. ananatis* has also been considered an emerging plant pathogen due to increasing reports of disease outbreaks in the previously undescribed host and geographical regions (Coutinho and Venter, 2009). This emergence is likely to have resulted from the persistent nature of *P. ananatis* in diverse environments through its association with a wide range of non-host plant and even insect vectors (Gitaitis et al., 2003; Dutta et al., 2014).

The virulence factors that have been identified as necessary for pathogenesis of *P. ananatis* in onion are motility for attachment (Weller-Stuart et al., 2016) and quorum sensing (QS) for production of biofilm and exopolysaccharide (EPS) (Morohoshi et al., 2007). In addition, genomic regions named “HiVir” (Asselin et al., 2018) and “Onion Virulence Region” (Stice et al., 2018), encoding enzymes catalyzing phosphonate biosynthetic pathway and cell wall degradation, respectively, have been characterized in the onion pathogenic strains of *P. ananatis*. For successful infection by *P. ananatis*, a rapid and collective expression of these virulence genes in response to the surrounding environment is critical as it results in the modulation of cellular pathways that predispose the pathogen for infection, pathogenesis, and survival in the host.

Hfq is an RNA-binding protein that constitutes a key component of post-transcriptional gene regulation exhibited by small non-coding regulatory RNAs (sRNAs) (Vogel and Luisi, 2011). Hfq is a ring-like homohexameric protein that was initially identified as a host factor needed for the replication of RNA bacteriophage Qβ (Franze de Fernandez et al., 1968). It is now known that the chaperone Hfq is essential for the structural stabilization of the class of *trans-*acting sRNAs whose regulatory mechanisms are dependent on Hfq (Updegrove et al., 2016). The chaperone facilitates imperfect base-pairing between the sRNA and its cognate messenger RNA (mRNA), forming an Hfq–sRNA–mRNA complex that determines the fate of target mRNA translation (Gottesman and Storz, 2011; Storz et al., 2011). Suppression of the protein synthesis is achieved by the formation of a sRNA–mRNA duplex at the 5′-untranslated region (UTR) of the transcript by occlusion of ribosome binding and/or by recruiting ribonucleases for mRNA degradation (De Lay et al., 2013). Conversely, translation of the mRNA is enhanced by Hfq–sRNA complexes that alter the 5′-secondary inhibitory structure of an mRNA, making it more accessible for initiation of translation.

Hfq-dependent sRNAs are typically 50–300 nucleotides in length and are *trans*-encoded from their cognate mRNAs. They are mostly found in, but not limited to, the intergenic regions of bacterial chromosomes (Argaman et al., 2001; Chao et al., 2012; Guo et al., 2014), and are characterized by often possessing a Rho-independent terminator at the 3′-end, resulting in a poly-uridine tail of sRNA that are recognized by Hfq (Otaka et al., 2011). The cellular functions modulated by Hfq–sRNAs are diverse, ranging from cell membrane integrity, acquisition, and metabolism of nutrients, motility, secretion systems, stress response, and virulence (Chao and Vogel, 2010). Their role in virulence regulation has been extensively studied in bacterial pathogens of animals. For example, in *Salmonella typhimurium*, motility and expression of the T3SS encoded within *Salmonella* pathogenicity island SPI-1 and SPI-2 are dependent on Hfq and contribute significantly to the adhesion and invasion of *Salmonella* into the host cells (Sittka et al., 2007, 2008) whereas in *Vibrio cholera*, Hfq and its *trans* acting sRNAs *Qrr* 1–4 regulate cholera toxin (CT) biosynthesis (Bardill and Hammer, 2012) and QS as an ultrasensitive switch to transition *V. cholerae* from low to high cell density mode for colonization and disease development (Lenz et al., 2004).

Despite the growing evidence of Hfq and Hfq-dependent sRNAs as a global post-transcriptional gene regulatory complex, the functionality of Hfq and its *trans* acting sRNAs in plant pathogenic bacteria has only been investigated in a few bacterial species to date, namely in *Agrobacterium tumefaciens* (Wilms et al., 2012a, b), *Burkholderia glumae* (Kim et al., 2018), *Dickeya dadantii* (Yuan et al., 2019), *Erwinia amylovora* (Zeng et al., 2013; Zeng and Sundin, 2014), *Pectobacterium carotovorum* (Wang et al., 2018), and *Xanthomonas* spp. (Schmidtke et al., 2013). Consequently, the functional role of Hfq and the diversity of Hfq-dependent sRNAs in phytopathogens remain largely elusive. We hypothesized that Hfq and Hfq-dependent sRNAs would play a critical role in *P. ananatis* pathogenesis, through direct regulation of specific virulence traits and through regulation of QS system. In this study, we functionally characterized the role of Hfq as a regulator in the production of acyl-homoserine lactones (AHLs), biofilm development, motility, and virulence, and identified the Hfq-dependent sRNAs that are potentially implicated in the regulation of the virulence traits of the ubiquitous plant pathogen *P. ananatis*.

## Materials and Methods

### Bacterial Strains and Growth Conditions

The bacterial strains and plasmids used in this study are listed in Table 1. *P. ananatis* LMG2665^T^ and *Escherichia coli* DH5α strains were cultured in Luria-Bertani (LB) broth [1% (w/v) NaCl, 1% (w/v) tryptone, and 0.5% (w/v) yeast extract; pH 7.2] or on LB agar plates [LB broth amended with 1.5% (w/v) agar; pH 7.2] at 28 and 37°C, respectively. The growth medium was supplemented with either ampicillin (100 μg/ml), chloramphenicol (50 μg/ml), gentamicin (20 μg/ml), or kanamycin (50 μg/ml) for plasmid DNA selection and maintenance.

**TABLE 1:** A list of strains or plasmids used in this study.

**Strain or plasmid**		**Characteristics^a^**	**Source**
Strains			
	*Escherichia coli* DH5α	F^–^ φ80*lac*ZΔM15 Δ(*lac*ZYA-*arg*F)U169 *rec*A1 *end*A1 *hsd*R17(r_K_^–^, m_K_^+^) *pho*A *sup*E44 λ^–^ *thi*-1 *gyr*A96 *rel*A1	Invitrogen
	*Chromobacterium violaceum* CV026	ATCC 31532 derivative, *cviI*:Tn*5xylE*; Km^r^, Sm^r^	McClean et al., 1997
Pantoea ananatis			
	LMG 2665^T^	Wild-type	Serrano, 1928
	LMG 2665^T^ (pRSFredTER)	LMG 2665^T^ transformed with pRSFredTER, Cm^r^	This study
	LMG 2665^T^ (pBBR1MCS-START-5)	LMG 2665^T^ transformed with pBBR1MCS-START-5, Gm^r^	This study
	LMG 2665^T^ Δ*hfq*	*hfq* deletion mutant, Km^r^	This study
	LMG 2665^T^ Δ*hfq*-pBBR1MCS-5_START::*hfq*	LMG 2665^T^ Δ*hfq* transformed with pBBR1MCS-START-5::*hfq*, Km^r^, Gm^r^	This study
Plasmids			
	pKD13	Broad-host range vector, mutagenesis cassette template, Km^r^	Datsenko and Wanner, 2000
	pRSFredTER	Broad-host range vector, expresses bacteriophage λ red recombinase (*bet*, *exo*, *gam*) and *sacB*, Cm^r^	Katashkina et al., 2009
	pBBR1MCS-START-5	Broad-host range vector, promoterless, Gm^r^	Obranić et al., 2013
	pBBR1MCS-START-5::*hfq*	pBBR1MCS-START-5 containing *hfq* and 613 bp upstream region (native promoter) cloned as *Sma*I and *Bam*HI, Gm^r^	This study

*^*a*^Cm^*r*^, Gm^*r*^, Km^*r*^, and Sm^*r*^ represent chloramphenicol, gentamicin, kanamycin, and streptomycin resistance, respectively.*

### Generation of a *P. ananatis hfq* Mutant and Complemented Strains

A mutant strain with chromosomal deletion of a single copy gene *hfq* (locus tag: PANA_RS17940) was constructed as previously described (Katashkina et al., 2009; Shyntum et al., 2015). The modification was made in the preparation of the knockout cassette which was amplified from the pKD13 plasmid using the Kan-F and Kan-R primers (Table 2) consisting of 50 bp homologous sequences of *hfq* flanking regions and 20 bp of kanamycin resistance gene priming sequences. Insertion of the kanamycin resistance gene was verified by Southern blotting, PCR amplification, and sequencing of the *hfq* region.

**TABLE 2:** A list of primers used in this study.

**Primer name**		**Sequence (5′–3′)**	**Length (nt)**
Mutagenesis			
	Kan-F	ACGTCGCTTATATAAAAAGACCAGGATGGAAAACCT GACGCTTTCCGATGCGATTGTGTAGGCTGGAGCT	70
	Kan-R	TTACGCAGTTTTTTTCAGAACCACTGTGTTCTACAA GCAACAAACAACAAATTCCGGGGATCCGTCGACC	70
	Test-F	TAGTGCGAAGCATGGGTG	18
	Test-R	TGCTCACCGGCATCATAACGG	21
	Southernblot-F	GCGATTGTGTAGGCTGGAGCT	21
	Southernblot-R	TCGGATGGAAGCCGGTCTTGTCG	23
Complementation			
	Comp-F	AAAAGGATCCGAGGCTGGGAGCTTTACATCG	31
	Comp-R	CGGTCAAACAAGCTATAACCTCG	23
qRT-PCR			
	ffh-F	CATTGAGATCAAAACCGTCG	20
	ffh-R	TGGGCGACGTGCTGTCGCT	19
	Arcz-F	GCAAGTGTTAACCAATACCC	20
	Arcz-R	GGGTGCGCTAATACTGC	17
	FnrS-F	GGTGAATGCAACGTCAA	17
	FnrS-R	GTTAGCCGGCGTATTTC	17
	GlmZ-F	CATAAACCTGGGAATGACG	19
	GlmZ-R	AGCAGGTGTAAGATCAGG	18
	RprA-F	TACCATGTTTCCTATGTTGG	20
	RprA-R	GATGGGCAAAGACTACAC	18
	RyhB2-F	TCGCGTGTCATCGACACGG	19
	RyhB2-R	GGCTGGCTAAATAATACTGGAAGC	24
	RyeB-F	CGAAAGCCTCTTATTAATGCC	20
	RyeB-R	AGACCGAACACGATTCC	17
	pPAR237-F	GTGGGAAAGCGAAGGTA	17
	pPAR237-R	CTTTCCGGCCAGACTTC	17
	pPAR238-F	CTGAAACAGCCAACACC	17
	pPAR238-R	GGATGTTACTCTGAGTGTCC	20
	pPAR395-F	TGGCGACAATTCAGATGG	18
	pPAR395-R	CCGCACCTCGTTAAAGG	17
5′-RACE			
	Linker nested-F	GAGGACACTGACATGGAGG	19
	FnrS-R	GTTAGCCGGCGTATTTC	17
	FnrS-nR	AGACAATATGGAGCGCAACG	20
	GlmZ-R	AGCAGGTGTAAGATCAGG	18
	GlmZ-nR	CGAGAGGTACCCGACTCAACGTG	23
	pPAR237-R	ACTTTCCGGCCAGACTTCACA	21
	pPAR237-nR	GGGACACTCAGAGTAACATCC	21
	pPAR238-R	TGAGTGTCCCGGCCAGCATCACT	23
	pPAR238-nR	TCCCTGGTGTTGGCTGTTTC	20
	pPAR395-R	CCGCACCTCGTTAAAGG	17
	pPAR395-nR	CCTAAATGACTTCCAAACAGCG	22

The promoter sequence of *hfq* determined in *E. coli* K12 MG1655 by Kim et al. (2012) was searched against the upstream sequence of *hfq* start codon in *P. ananatis* LMG2665^T^. An amplicon (1038 bp) containing the *hfq* gene (315 bp), its native promoter (58 bp), and flanking sequences (662 bp) was cloned into a pBBR1MCS-5_START vector (Obranić et al., 2013) restricted with *Sma*I and *Bam*HI enzymes. Electrocompetent *hfq* deletion mutant *P. ananatis* was transformed with *hfq* complementing plasmid, pBBR1MCS::*hfq* and the resulting transformants were selected on the gentamicin amended LB agar. The integrity of the *hfq* complementation was determined by plasmid extraction, PCR, and sequencing using Test-F and Test-R primers (Table 2).

### *In vitro* and *in planta* Growth Assay

The growth of wild-type *P. ananatis* with an empty pBBR1MCS-5_START vector (WT), *hfq* deletion mutant with an empty pBBR1MCS-5_START vector (Δ*hfq*), and *hfq* complementing pBBR1MCS-5_START::*hfq* (pBBR1MCS::*hfq*) strains of *P. ananatis* was monitored both *in vitro* and *in planta* conditions.

*Pantoea ananatis* strains grown overnight in LB broth were normalized to an OD_600__nm_ reading of 0.5. For *in vitro* growth assay, the normalized cultures were diluted 100-fold in fresh LB medium and incubated with shaking at 200 rpm. The absorbency of each culture was periodically measured. There were three replicates for each culture and the experiment was repeated twice.

The previously described red onion scale assay (Stice et al., 2018) was adapted for quantifying *in planta* growth of *P. ananatis* between the WT, Δ*hfq*, and *hfq* complementing strains. In summary, sliced red onion (*Allium cepa* L) scales of approximately 9 cm^2^ in area were surface sterilized in 3% bleach solution for 1 min and were rinsed twice in distilled water. Each scale was inoculated with 1 μl of bacterial cells (1 × 10^7^ CFU/ml) suspended in 1× PBS [0.8% (w/v) NaCl, 0.02% (w/v) KCl, 0.144% (w/v) NaHPO_4_, 0.024% KH_2_PO_4_; pH 7.4] using a sterile pipette tip. Inoculated scales were placed on moistened paper towels in a surface sterilized container and were incubated at room temperature for 5 days. To quantify growth, three onion scales per strain were harvested at 24 h intervals. Each scale was macerated in 1 ml of 1× PBS and the extract was serially diluted and cells were enumerated on LB supplemented with gentamicin. Experiments were repeated in triplicate, and the results were presented as CFU/g of onion tissue. Sterile water was used as a negative control.

### Virulence Assay

Virulence assay was performed as previously described for *in planta* growth assay. The vertical diameter of the water-soaked lesion on onion scales inoculated with WT, Δ*hfq*, and *hfq* complementing *P. ananatis* strains was measured at 3 days post inoculation (dpi). The virulence assay was repeated twice, and there were three technical replicates for each *P. ananatis* strains.

### Motility Assay

Overnight cultures of *P. ananatis* strains (WT, Δ*hfq*, and pBBR1MCS::*hfq*) were normalized to OD_600__nm_ = 0.5 and 1 μl of each culture was inoculated in the center of the soft agar [0.5% (w/v) NaCl, 1% (w/v) tyrptone, and 0.3% (w/v) agar; pH 7.2]. The inoculated plates were incubated at 28°C, and swimming motility was determined after 24 h. Negative control plates were inoculated with sterile water. The swimming motility experiment was repeated three times with three biological replicates in each experiment.

### Bioassay Detection of Acyl-Homoserine Lactones

Formation of AHL by WT, Δ*hfq*, and *hfq* complementing strains of *P. ananatis* was determined using experimental procedures adapted from McClean et al. (1997). An aliquot (0.5 ml) of AHL reporter strain *Chromobacterium violaceum* (*C. violaceum* 026) grown in LB overnight was spread plated on LB agar plates and air-dried. Thereafter, three wells were (three replicates) created on each plate by puncturing the agar with a sterile cork-borer and inoculated with 100 μl of cell-free filtrate of *P. ananatis* WT, Δ*hfq*, and *hfq* complementing strains overnight cultures. The inoculated plates were incubated at 28°C for 48 h. The formation of violacein (purple halo) by CV026, around the inoculated wells were indicative of AHL production. The assay was repeated twice and carried out in three technical replicates.

### Biofilm Quantification

The biofilm of WT, Δ*hfq*, and *hfq* complementing strains of *P. ananatis* was quantified as previously described by Santander and Biosca (2017) with slight modifications. An aliquot of 160 μl broth culture diluted to an OD_600__nm_ of 0.5 in half-strength LB [0.5% (w/v) NaCl, 0.5% (w/v) tryptone, and 0.25% (w/v) yeast extract; pH 7.2] was made into each well of a polystyrene 96-well microplate (Nunc^TM^ MicroWell^TM^, Thermo Scientific, Waltham, MA, United States) and incubated for 24 h under static conditions. Eight replicates per *P. ananatis* strain were included in each experiment with sterile half-strength LB broth serving as a negative control. Thereafter, the inoculated 96-well plates were inverted to remove the excess LB broth, air-dried, and incubated at 60°C for 40 min to heat-fix the biofilms. The biofilms were stained with 1% crystal violet (220 μl) for 15 min before being rinsed with distilled water. After rinsing and invert-air-drying the microplate, 220 μl of ethanol:acetone in 8:2 ratio was added to the wells to solubilize the crystal violet dye for 20 min at room temperature. The solubilized biofilm was measured at OD_600_ using Safire Microplate Reader (Tecan, Research Triangle Park, NC, United States), and this assay was repeated three times.

### RNA Extraction and Transcriptomic Analysis

Total RNA of *P. ananatis* WT and Δ*hfq* strains grown in LB broth was extracted at OD_600__nm_ readings of 0.2 (T1 = low cell density) and 0.6 (T2 = high cell density) using the miRNeasy Mini kit (Qiagen, Hilden, Germany). Genomic DNA was removed by including an on-column DNase digestion step during the RNA extraction. The purity (A260/A280) of extracted RNA was measured by Nanodrop2000 (Thermo Scientific, Sugarland, TX, United States) and RNA integrity was determined by Agilent2100 Bioanalyzer (Agilent Technologies, Santa Clara, CA, United States). Illumina Truseq Small RNA Library (Illumina, San Diego, CA, United States) preparation was performed on the RNA samples, and deep sequencing of the library was conducted on Illumina HiSeq2500 platform (single-end, 1 × 50 bp) by Macrogen (South Korea).

### Bioinformatic Analysis and sRNA Identification

Raw sequencing reads (BioProject accession number: PRJNA550544) were stringently trimmed and filtered using Trimmomatic (Bolger et al., 2014) to remove adapter sequences and low quality reads. Following adapter trimming and filtering, quality was verified using FastQC (Andrews, 2010) and reads were mapped to the *P. ananatis* LMG20103 genome (De Maayer et al., 2010) using Bowtie2 (Langmead and Salzberg, 2012), as the genome of LMG 20103 was the only *P. ananatis* genome with a complete annotation at the time of analysis. For sRNA identification, a custom python script (Supplementary Data Sheet S1, see the section “genic_filter.py” in the Supplementary Material) was compiled to remove reads that mapped to coding sequences, ribosomal RNA, and transfer RNA, or within 120 bases upstream or downstream of these features from the resulting sequence alignment map (SAM) files. The purpose of the 120 base buffer was to reduce the number of sRNAs identified that originated from extended 5′- or 3′-UTR regions. All wild-type sequencing replicates from the same sampling time point were merged into a single gene-filtered SAM file for sRNA identification.

To identify putative sRNAs from gene-filtered SAM files, a custom python script (Supplementary Data Sheet S1, see the section “peak_ID.py” in the Supplementary Material) was used to calculate per base depth relative to the genome-wide per-base sequencing depth by replicate, which was also normalized to library size. A threshold of 10-fold increased abundance above background with a minimum length of 10 nucleotides was chosen for sRNA identification. Using the script, putative sRNAs at the low cell density and high cell density sampling time points were identified and the lists of sRNAs were merged using a custom python script (Supplementary Data Sheet S1, see the section “mergeList.py” in the Supplementary Material), combining any overlapping identified sRNAs into a single sRNA to generate a single list of putative *P. ananatis* sRNAs (pPARs sRNA).

### Computational Prediction of Rho-Independent Terminators

Following established criteria (Zeng and Sundin, 2014), Rho-independent terminators were searched in the *P. ananatis* LMG20103 genome using a custom python script (Supplementary Data Sheet S1, see the section “RI_term.py” in the Supplementary Material). Briefly, the search was conducted in an effort to detect poly-T regions with at least six continuous Ts and for those that had at least four GC base pairs in the last six bases before the poly-T stretch. Of these, those that had at least 50% GC content in the last 25 bases before the poly-T were considered to be putative Rho-independent terminators.

### Differential sRNA Expression in *P. ananatis* LMG 2665 WT vs. δ*hfq*

Using the genomic coordinates from the BLAST + search of the pPAR sRNAs against the *P. ananatis* LMG2665 genome (Adam et al., 2014), a gene format file (.gff) for all the pPAR sRNAs was generated. The sRNA sequencing reads that had been trimmed and filtered were mapped to the LMG2665 genome using Bowtie2 (Langmead and Salzberg, 2012). The mapped reads were sorted using SAMtools (Li et al., 2009) and the number of reads mapping to pPAR sRNAs in the LMG2665 genome was counted using HTSeq (Anders et al., 2015). Read counts tables were analyzed for statistically significant differential expression of pPAR sRNAs between WT- and δ*hfq-*mutant samples at corresponding time points using the DESeq R package which utilizes a negative binomial distribution model (Anders and Huber, 2010; R Core Team, 2013). Resulting genes with a false-discovery rate of 0.05 were considered differentially expressed.

### sRNA Conservation Analysis

The bacterial genomes were downloaded from NCBI and searched using BLAST+ (Camacho et al., 2009) with all pPAR sRNA sequences as queries. Because BLAST uses local alignment, the global percent identity to pPAR sRNAs was calculated by multiplying the percent identity by the length of the BLAST alignment and dividing by the length of the pPAR sRNA. Heatmaps showing percent identity of sRNA by genome were generated using ClustVis (Metsalu and Vilo, 2015).

### qRT-PCR Validation of sRNA Expression

To validate the expression of putative sRNAs identified, a quantitative RT-PCR was conducted on a subset of sRNAs. The 2 μg of total RNA extracted from the two time points (low and high cell density) was converted to cDNA using random primers using the High-Capacity cDNA Synthesis Kit (Applied Biosystems, Carlsbad, CA, United States). Subsequently, PowerUP^TM^ SYBR^TM^ Green Master Mix (Applied Biosystems, Carlsbad, CA, United States) was used to quantify expression levels of the selected sRNAs real time in QuantStudio 12K Flex Real-Time PCR System (Applied Biosystems, Carlsbad, CA, United States). The list of primers used for qRT-PCR is found in Table 2. The relative expression of sRNA was calculated using 2^–Δ^ ^Δ^ ^CT^ method (Livak and Schmittgen, 2001) with the gene *ffh* encoding a signal recognition particle protein, serving as an endogenous mRNA control (Takle et al., 2007; Sibanda et al., 2018).

### 5′-Rapid Amplification cDNA Ends Analysis

The 5′-Rapid Amplification cDNA Ends (RACE) analysis was conducted on the selected putative sRNAs to capture their transcription start sites (TSS). Total RNA (up to 15 μg) of *P. ananatis* strains grown to high density (OD_600_ = 0.6) was extracted as above mentioned (see the section “RNA Extraction and Transcriptomic Analysis”). The resulting RNA was ligated to 300 pmol of RNA linker: GACGAGCACGAGGACACUGACAUGGAGGAGGGAGUAG AAA in the presence of RNA 5′-pyrophosphohydrolase (RppH) (New England BioLabs, Ipswich, MA, United States) and T4 RNA ligase (New England BioLabs, Ipswich, MA, United States) at 37°C for 4 h. The linker-ligated RNA was purified using Trizol-chloroform (2:1) extraction method, as described by Rio et al. (2010). The resulting RNA was ethanol precipitated and suspended in 10 μl of RNase-free water. The cDNA of linker-ligated RNA was synthesized as previously described (see the section “qRT-PCR Validation of sRNA Expression”) and Gene Specific PCR (GSP) was performed using nested linker and sRNA-specific PCR primers (Table 2). The GSP using genomic DNA was used as a control and resulting bands from 5′-RACE were gel-purified and cloned into pJET1.2 blunt (Thermo Fischer Scientific Baltics UAB, Vilnius, Lithuania) prior to sequencing.

### Secondary Structure and mRNA Target Prediction

The secondary structures of sRNAs, of which their TSS have been determined by 5′-RACE analysis, were predicted *in silico* using RNAfold web server^1^ (Hofacker, 2003). The putative target mRNAs of novel sRNAs pPAR237, pPAR238, and pPAR395 and their putative interacting domains were computationally predicted using CopraRNA and IntaRNA^2^ (Wright et al., 2014). The above information is presented in Supplementary Figure S6 and Supplementary Table S4, respectively.

### Image and Statistical Analysis

Images resulting from motility, AHL detection, and virulence assays were analyzed in ImageJ (Schneider et al., 2012) for measurement of halos and lesion diameter. Statistical analyses are performed with R 3.2.6 (R Core Team, 2013) and significance of the data (*P* < 0.05) were determined by analysis of variance (ANOVA) and Tukey’s honestly significantly difference (HSD) tests. Except where otherwise mentioned, all data shown in this study represent mean values and error bars represent standard error (SE) of the samples.

## Results

### hfq Mutation Negatively Affects Growth

To investigate the functional role of Hfq in the pathogenesis of *P. ananatis*, an *hfq* deletion mutant (Δ*hfq*) was constructed by replacing the *hfq* gene with a kanamycin resistance marker (the section “Materials and Methods”). Southern blotting (Supplementary Figure S1) and PCR amplification of the *hfq* region (Supplementary Figure S2) verified a single insertion of the antibiotic marker in the *hfq* mutant strain. For the construction of the *hfq-*complementing plasmid, *hfq* promoter sequence of *E. coli* K12 was used to search for *hfq* promoter in *P. ananatis* and a highly conserved *hfq* promoter sequence (93% nucleotide identity) of *P. ananatis* compared to that of *E. coli* K12 was found overlapping in the coding region of the adjacent gene *miaA*.

*In vitro* growth analyses of *P. ananatis* WT, Δ*hfq*, and *hfq* complementing strains cultured in LB medium showed that the *hfq* mutation affected the growth of *P. ananatis*. The *hfq* mutant exhibited a slower growth rate relative to the WT and *hfq*-complementing strains, but similar cell density was reached at stationary phase as the both strains (Supplementary Figure S3). Similarly, *in planta* growth curves at 12 h showed that WT, Δ*hfq*, and *hfq* complementing strains of *P. ananatis* exhibited comparable cell densities to one another (Supplementary Figure S4B) which were at sufficient levels for the onset of symptoms by 3 dpi (Supplementary Figure S4A).

### Loss of Hfq Attenuates Virulence in *P. ananatis*

**FIGURE 1:**
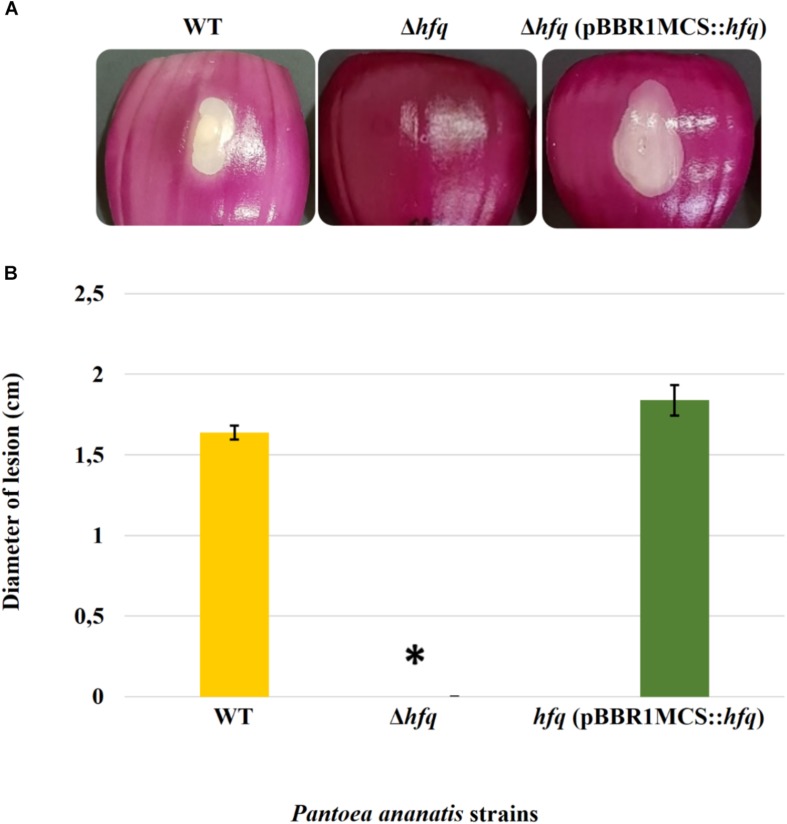
Effect of deletion of *hfq* on the virulence of *P. ananatis.* **(A)** Water-soaked lesion caused by the wild-type (WT), *hfq* mutant (Δ*hfq*), and *hfq* complementing [Δ*hfq* (pBBR1MCS::*hfq*)] strains of *P. ananatis* LMG 2665^T^ in red onion scales at 3 days post inoculation (dpi). **(B)** The diameter of the lesion caused by the WT, Δ*hfq*, and Δ*hfq* (pBBR1MCS::*hfq*) strains was measured at 3 dpi from three replicates for each strain. Mean lesion length from two independent assays is plotted. An asterisk denotes significance differences (*P* < 0.05) in the lesion size caused by Δ*hfq* relative to WT *P. ananatis*.

Virulence assay on red onion scales demonstrated clearing of the red pigment and formation of a water-soaked lesion in the onion scales inoculated with WT *P. ananatis* while no disease symptoms were observed on the scales infected with the *P. ananatis* Δ*hfq* mutant (Figure 1). The impaired virulence of the *P. ananatis* Δ*hfq* mutant was restored to the wild-type levels by *trans* expression of *hfq* gene on the plasmid pBBR1MCS-5_START::*hfq*. The finding that the *P. ananatis* Δ*hfq-*mutant strain was able to attain *in planta* population densities equivalent to WT (Supplementary Figure S4) suggests that the lack of disease symptoms is not associated with a growth defect, and that *hfq* is required for virulence of this strain when inoculated into red onion.

### Hfq Regulates Motility, AHL Production, and Biofilm Formation

**FIGURE 2:**
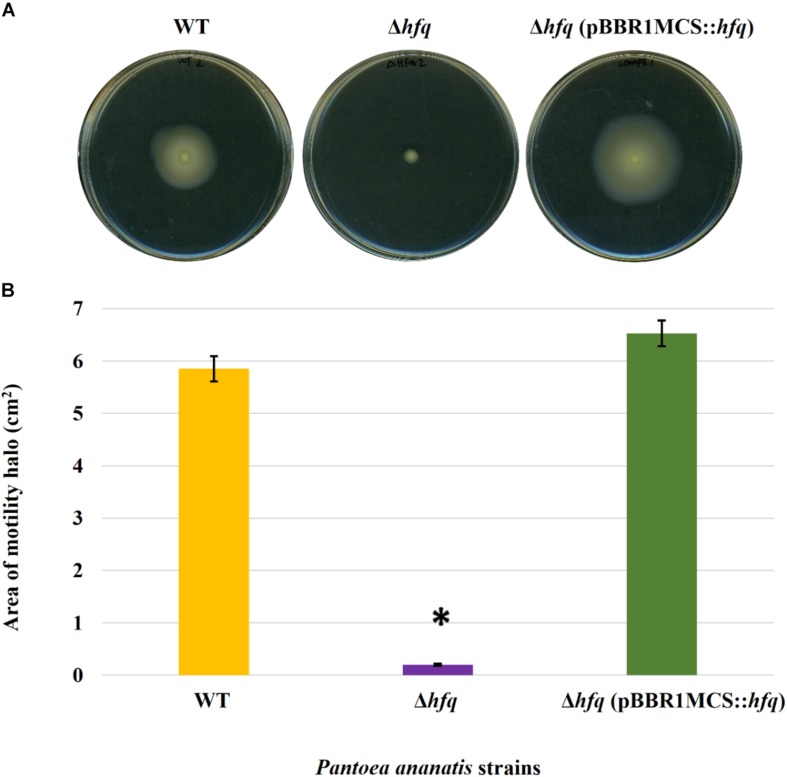
Effect of deletion of *hfq* on the motility of *P. ananatis*. **(A)** Motility of wild-type (WT), *hfq* mutant (Δ*hfq*), and *hfq* complementing (Δ*hfq* pBBR1MCS::*hfq*) strains of *P. ananatis* LMG 2665^T^ after 24 h on 0.3% swimming agar plates. **(B)** Motility area of WT, Δ*hfq*, and Δ*hfq* (pBBR1MCS::*hfq*) strains was measured at 24 hpi. An asterisk denotes significant differences (*P* < 0.05) in the motility of Δ*hfq* relative to WT *P. ananatis*.

**FIGURE 3:**
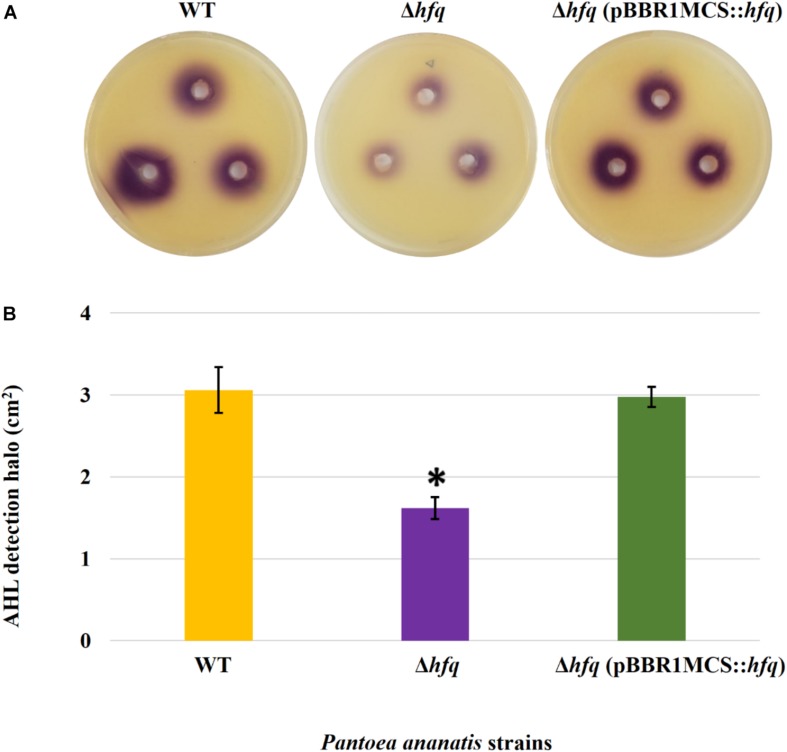
AHL detection bioassay. **(A)** Detection of AHLs produced by the wild-type (WT), *hfq* mutant (Δ*hfq*), and *hfq* complementing [Δ*hfq* (pBBR1MCS::*hfq*)] strains of *P. ananatis* LMG 2665^T^ by biosensor *Chromobacter violaceum* CV026 in the form of a purple (violacein) halo after 48 h. **(B)** Mean areas of the violacein halo (excluding the area of the well) from two independent experiments were measured for the WT, Δ*hfq*, and Δ*hfq* (pBBR1MCS::*hfq*) strains. Each plate contained three replicates. An asterisk denotes significant differences (*P* < 0.05) in the size of violacein halo of Δ*hfq* relative to WT *P. ananatis.*

**FIGURE 4:**
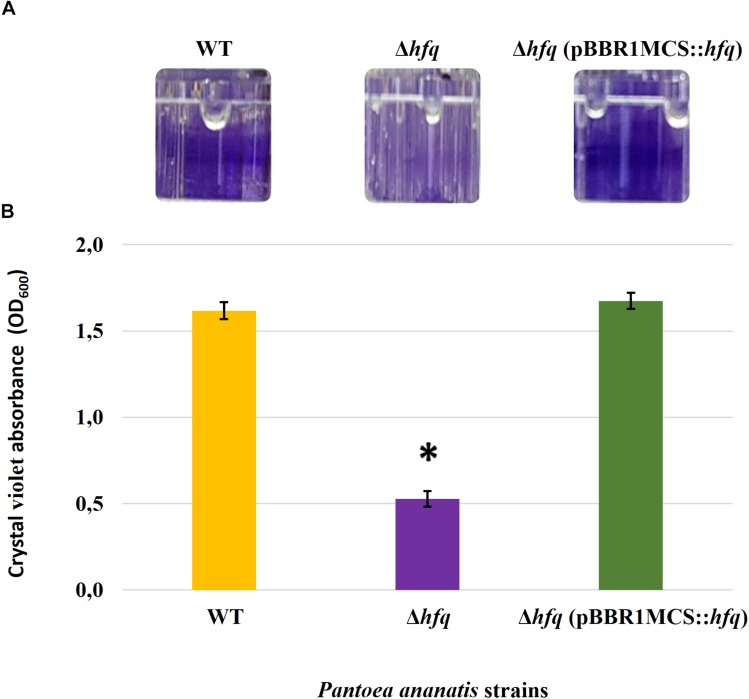
Effect of deletion of *hfq* on the biofilm-forming ability of *P. ananatis.* **(A)** Biofilms formed by the wild-type (WT), *hfq* mutant (Δ*hfq*), and *hfq* complementing [Δ*hfq* (pBBR1MCS::*hfq*)] strains of *P. ananatis* LMG 2665^T^ in 96-well microtiter plate after 24 h incubation under static conditions. **(B)** Quantification of the biofilms formed by the (WT), Δ*hfq*, and Δ*hfq* (pBBR1MCS::*hfq*) strains after 24 h using crystal violet (1%) staining method. The absorbance of solubilized biofilms stained with crystal violet was measured at an optical density wavelength of 600 nm. An asterisk denotes significance differences (*P* < 0.05) in the amount of biofilm formed by Δ*hfq* relative to WT *P. ananatis*.

To determine whether the *P. ananatis* Hfq regulates virulence traits, swimming motility, production of AHL molecules, and biofilm were quantified in WT, Δ*hfq*, and *hfq*-complementing strains of *P. ananatis*. The results show that *P. ananatis hfq* mutant was impaired in swimming motility relative to the wild-type strain, as determined by the size of the halo that formed on the soft agar (Figure 2). In addition, AHL production, as determined by the production of the purple pigment violacein by the *C. violaceum* 026 biosensor demonstrated a statistically significant reduction in the size of the purple halo formed by the *hfq*-mutant strain relative to the wild type, indicating a significant reduction in AHL production by the mutant strain (Figure 3). Furthermore, a threefold reduction (*P* < 0.05) in the biofilms formed by the *hfq*-mutant strain relative to the WT *P. ananatis* was also observed (Figure 4). These findings are consistent with previous studies that showed that AHL molecules are needed as a signal for QS to regulate biofilm formation in *P. ananatis* (Morohoshi et al., 2007; Sibanda et al., 2016). The phenotypic defects resulting from loss of *hfq*, which were restored to wild-type levels by *trans*-complementation of *hfq*, suggest that Hfq regulates the production of multiple virulence traits in *P. ananatis*.

### Identification of Putative sRNAs

Due to impaired motility, AHL production, biofilm formation, and virulence caused by the loss of Hfq in *P. ananatis* LMG 2665, a sRNA sequencing analysis was conducted to identify the regulatory sRNAs that are dependent on Hfq for stability and function. Deep sequencing of the sRNA transcriptomes of WT and Δ*hfq-*mutant *P. ananatis* strains at low (OD_600_ = 0.2) and high cell density (OD_600_ = 0.6) time points resulted in a total of 172.03 million reads. Following trimming of adapters and filtering for high-quality reads (Phred score =30), 66.74 million reads were retained, of which 83.2% mapped uniquely to the *P. ananatis* LMG20103 genome. Following removal of the reads that mapped to protein coding genes, rRNAs, or tRNAs, 9.72 million reads remained for the sRNA identification and analysis. The distribution of reads across the WT and Δ*hfq-*mutant *P. ananatis* strains, each with three technical replicates at low and high cell density time points, are included in Supplementary Table S1.

For identification of sRNAs in the transcriptome dataset, the WT sequencing data were utilized and calculated for the per-base depth across the genome relative to the genome-wide average per-base depth. To select a threshold that would allow for sensitive detection of sRNAs while also filtering out noise in the sequencing data, the number of putative sRNAs identified across a broad range of signal-to-noise thresholds was calculated. A strong linear relationship (*R*^2^ = 0.9981) between Log_10_ (Threshold) and Log_10_ (# sRNAs identified) was found (Supplementary Figure S5), and a signal-to-noise threshold ratio of 10 was selected for calling of putative sRNAs from the sequencing data. Using this threshold, a total of 615 pPARs sRNAs was identified. Of these, 425 pPARs were identified in both time points, 90 were identified only in the low cell density time point, and 100 only in the high cell density time point in *P. ananatis* LMG2665.

### Characterization of pPAR sRNAs

**FIGURE 5:**
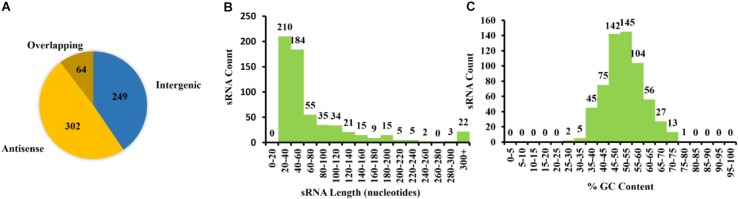
Characterization of *Pantoea ananatis* sRNAs (pPAR sRNA). **(A)** A total of 615 putative *P. ananatis* LMG 2665^T^ sRNAs were classified into 302 antisense, 64 overlappings, and 249 intergenic pPAR sRNAs. **(B)** The mean length of pPAR sRNAs was 66.4 bases with a median of 42 bases. **(C)** The mean GC content of pPAR SRNAs was 52.3% with a median of 52.2%.

The 615 identified pPARs were further classified as intergenic, antisense, or overlapping. The classification resulted in 249 intergenic pPAR sRNAs, 302 antisense pPAR sRNAs, and 64 overlapping pPAR sRNAs (Figure 5A). The mean length of pPAR sRNAs was 66.4 bases with a median of 42 bases (Figure 5B) and mean GC content of pPAR SRNAs was 52.3% with a median of 52.2% (Figure 5C). Both of these are quite close to the genome average of 53.7% GC bases (De Maayer et al., 2010). Of note, seven pPAR sRNAs had GC content below 30%, and 14 pPAR sRNAs had GC content above 70%, suggesting the potential horizontal acquisition of the genomic regions containing these sRNAs.

In addition, we performed a genome-wide computational search for putative Rho-independent terminators that are associated with the transcription termination of Hfq-dependent sRNAs (Otaka et al., 2011). The results revealed that there were 5,002 poly-T stretches with at least 6 continuous Ts and 2,437 of these had four or more GC base pairs in the last 6 bases before the poly-T. A total of 1,842 of poly-T stretches had approximately 50% GC content in the final 25 bases before the poly-T, meeting the established criteria of Rho-independent terminators (Zeng and Sundin, 2014). Based on these criteria, only 569 were associated with protein-coding genes and 69 were associated with the identified pPAR sRNAs. The key features of select pPAR sRNAs are presented in Table 3 and for full data, including genomic coordinates and sRNA sequences, refer to Supplementary Table S2.

**TABLE 3:** A list of selected *Pantoea ananatis* LMG2665^T^ pPAR sRNAs.

**sRNA locus_ID**	**sRNA name**	**Strand^a^**	**Start^a^**	**End^a^**	**Length (nt)**	**Classification**	**RI-terminator^b^**	**Hfq-dependent**
pPAR009	–	+	97939	98052	113	Intergenic	Yes	Yes
pPAR026	glmZ	+	183885	184074	189	Intergenic	Yes	Yes
pPAR035	–	−	241229	241262	33	Antisense	Yes	No
pPAR052	–	+	318391	318427	36	Intergenic	Yes	Yes
pPAR063	arcZ	−	497228	497409	181	Intergenic	Yes	Yes
pPAR069	–	+	518137	518185	48	Overlapping	Yes	Yes
pPAR091	–	+	758889	758998	109	Intergenic	Yes	No
pPAR143	–	+	1192217	1192317	100	Overlapping	Yes	Yes
pPAR155	–	+	1306802	1306953	151	Antisense	Yes	Yes
pPAR165	–	−	1480479	1480511	32	Intergenic	Yes	No
pPAR184	–	−	1706868	1706902	34	Antisense	Yes	No
pPAR204	–	−	1900966	1901000	34	Antisense	Yes	No
pPAR205	rprA	−	1918007	1918133	126	Intergenic	Yes	Yes
pPAR237	–	−	2187335	2187526	191	Intergenic	Yes	Yes
pPAR241	–	−	2226977	2227034	57	Intergenic	Yes	Yes
pPAR245	fnrS	−	2254671	2254792	121	Intergenic	Yes	Yes
pPAR246	ryhB	−	2267249	2267331	82	Intergenic	Yes	Yes
pPAR263	–	+	2451765	2451968	203	Antisense	Yes	No
pPAR287	–	+	2750610	2750660	50	Intergenic	Yes	Yes
pPAR307	–	+	2914323	2914402	79	Intergenic	Yes	Yes
pPAR330	–	−	3095161	3095223	62	Intergenic	Yes	No
pPAR332	–	−	3146708	3146885	177	Intergenic	Yes	Yes
pPAR337	–	−	3235861	3235937	76	Antisense	Yes	Yes
pPAR343	ssrA	+	3274707	3274936	229	Intergenic	Yes	Yes
pPAR345	–	+	3335859	3335962	103	Overlapping	Yes	Yes
pPAR364	–	+	3475041	3475225	184	Intergenic	Yes	No
pPAR367	–	−	3505014	3505042	28	Antisense	Yes	No
pPAR388	–	+	3747994	3748044	50	Antisense	Yes	No
pPAR394	–	−	3822243	3822284	41	Intergenic	Yes	Yes
pPAR395	–	−	3822866	3822977	111	Intergenic	Yes	Yes
pPAR404	–	+	3929664	3929700	36	Antisense	Yes	No
pPAR418	–	+	4001816	4001864	48	Overlapping	Yes	No
pPAR433	ryhB	−	4105322	4105419	97	Intergenic	Yes	Yes
pPAR442	–	−	4239633	4239719	86	Intergenic	Yes	No
pPAR447	–	+	4256795	4256882	87	Intergenic	Yes	No
pPAR457	–	−	4330842	4330892	50	Overlapping	Yes	Yes
pPAR463	–	−	4372873	4372966	93	Intergenic	Yes	No
pPAR464	spf	−	4373881	4373992	111	Intergenic	Yes	Yes
pPAR470	–	−	4388229	4388352	123	Overlapping	Yes	Yes
pPAR479	–	−	4457282	4457307	25	Intergenic	Yes	Yes
pPAR509	–	+	122956	123006	50	Intergenic	Yes	No
pPAR511	–	−	132823	132844	21	Antisense	Yes	Yes
pPAR521	–	+	392904	392975	71	Antisense	Yes	No
pPAR525	–	−	477597	477633	36	Intergenic	Yes	No
pPAR535	–	+	809520	809548	28	Antisense	Yes	Yes
pPAR544	–	+	900462	900550	88	Antisense	Yes	No
pPAR582	–	+	2552244	2552278	34	Intergenic	Yes	No
pPAR590	–	−	2886291	2886321	30	Antisense	Yes	Yes
pPAR608	–	+	3962774	3962823	49	Antisense	Yes	Yes
pPAR626	–	+	4293304	4293392	88	Intergenic	Yes	No
pPAR632	–	+	4459587	4459632	45	Antisense	Yes	No
pPAR638	–	+	58679	58728	49	Intergenic	Yes	Yes
pPAR642	–	−	116116	116179	63	Overlapping	Yes	No
pPAR667	–	+	812084	812142	58	Overlapping	Yes	Yes
pPAR679	–	+	1035446	1035471	25	Intergenic	Yes	Yes
pPAR699	−	−	1501874	1501915	41	Antisense	Yes	Yes
pPAR714	–	−	2049786	2049816	30	Antisense	Yes	Yes
pPAR719	–	−	2171734	2171781	47	Intergenic	Yes	Yes
pPAR724	–	+	2341919	2341964	45	Antisense	Yes	Yes
pPAR726	–	−	2353498	2353521	23	Intergenic	Yes	Yes
pPAR732	sdsR/ryeA	−	2435078	2435148	70	Intergenic	Yes	Yes
pPAR765	–	−	3763787	3763821	34	Antisense	Yes	Yes
pPAR793	–	−	4420191	4420213	22	Intergenic	Yes	Yes
pPAR796	–	−	4502685	4502709	24	Intergenic	Yes	Yes

*^*a*^Based on location and position in genome of strain LMG20103 (2665 co-ordinates are found in Supplementary Table S1). ^*b*^RI terminator indicates the presence of a Rho-independent terminator sequence downstream of the sRNA sequence.*

### Identification of Hfq-Dependent sRNAs

**FIGURE 6:**
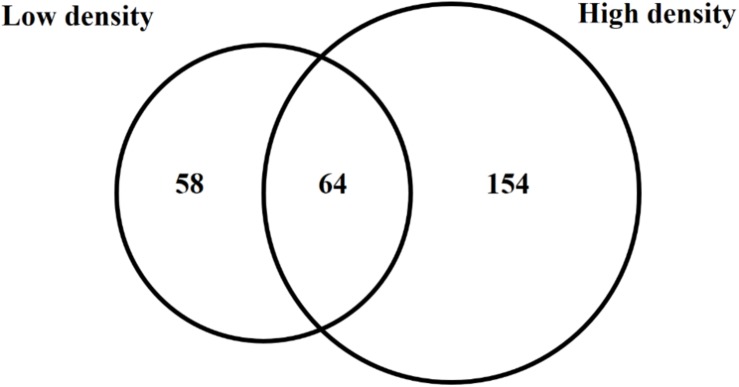
Hfq regulates several *Pantoea ananatis* sRNAs (pPAR sRNA). Venn-diagram of differentially expressed pPAR sRNAs between by the wild-type (WT) and *hfq* mutant (Δ*hfq*) *P. ananatis* LMG 2665^T^ grown *in vitro* at low (OD_600nm_ = 0.2) or high cell density (OD_600nm_ = 0.6).

Because *trans* encoded sRNAs have been shown to depend on RNA chaperone proteins such as Hfq for stability and activity (Vogel and Luisi, 2011), an *hfq* mutant was included in the sRNA sequencing experiment in order to determine pPAR sRNAs that are dependent on or influenced by the loss of *hfq.* The analysis of *hfq* to WT samples from both low cell density and high cell density samples identified a total of 276 pPAR sRNAs affected in abundance by Hfq. Sixty-four pPAR sRNAs were affected in abundance by loss of *hfq* in both cell density samples, 58 pPAR sRNAs in low cell density samples, and 154 pPAR sRNAs only in high cell density samples (Figure 6). Of all the Hfq-dependent pPAR sRNAs, 145 had decreased abundance and 131 had increased abundance in the *hfq* mutant relative to wild type. Overall, results indicate that Hfq affects the abundances of numerous pPAR sRNAs either positively and/or negatively. Supplementary Table S3 lists all pPAR sRNAs affected in abundance by loss of *hfq* as well as corresponding fold changes in both low and high cell densities.

**FIGURE 7:**
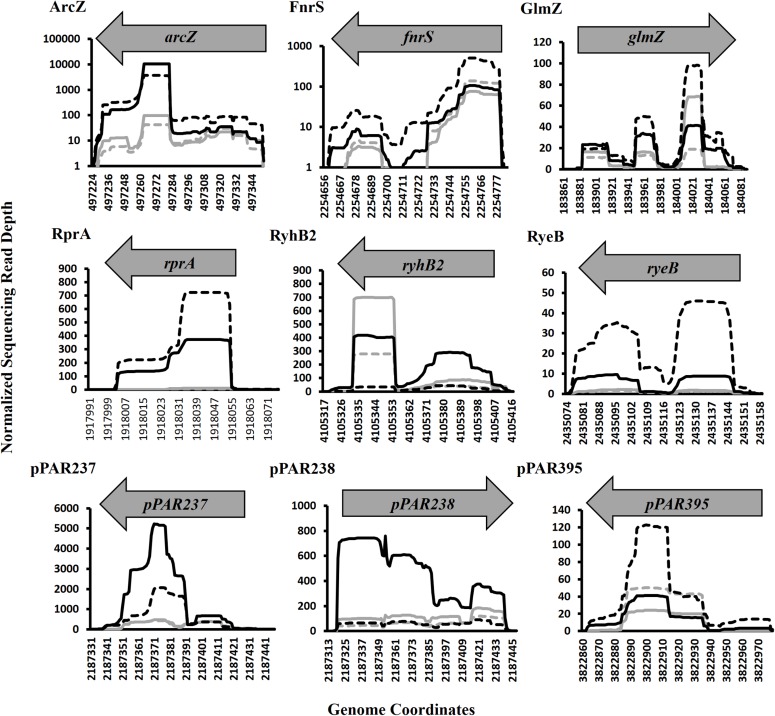
Sequencing read depth plots for selected *Pantoea ananatis* sRNAs (pPAR sRNA). Per-base sequencing read depth across the length of sRNAs, normalized to the genome-wide average per-base read depth was plotted for selected *P. ananatis* LMG 2665^T^ sRNAs. Solid black lines represent sRNA sequencing depth in wild-type (WT) *P. ananatis* at low cell density (OD_600nm_ = 0.2) and dashed black lines represent sRNA sequencing depth in WT at high cell density (OD_600nm_ = 0.6). Solid gray lines represent sRNA sequencing depth in *hfq* mutant *P. ananatis* (Δ*hfq*) at low cell density (OD_600nm_ = 0.2) and dashed gray lines represent sRNA sequencing depth in Δ*hfq* at high cell density (OD_600nm_ = 0.6).

Of the pPAR sRNAs affected by the loss of *hfq*, 41 have predicted Rho-independent terminators. Of these, 25 are intergenic and 16 are antisense, consistent with the classical model that Hfq-dependent sRNAs are frequently intergenic (Vogel and Luisi, 2011). Among the sRNAs detected in intergenic regions and Hfq-dependent with Rho-independent terminator, 9 known sRNAs and 16 novel sRNAs were identified. The known sRNAs included ArcZ, FnrS, GlmZ, RprA, RyeB/SdsR, RyhB, RyhB2, Spot42, and SsrA. The depth plots for a number of selected known and novel pPAR sRNAs of interest were generated, showing per-base sequencing depth across the length of the sRNA (Figure 7). Several pPAR sRNAs have certain regions

with far greater sequencing depth than the rest of the sRNA which suggests that mechanisms such as post-transcriptional processing (Davis and Waldor, 2007; Chao et al., 2017) might be active in *P. ananatis*, playing a role in sRNA maturation and/or processing.

### Conservation of Identified sRNAs

**FIGURE 8:**
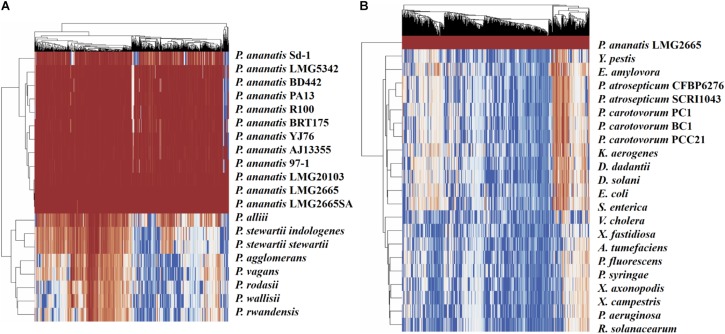
Conservation of *Pantoea ananatis* LMG 2665^T^ sRNAs (pPAR sRNAs) in genomes of **(A)** *Pantoea* species and **(B)** bacterial species outside of the genus *Pantoea*. BLAST+ was used to query each genome with each pPAR sRNA. Heatmap scale from 0 to 100 represents percent identity between the best BLAST hit and the *P. ananatis* pPAR sRNA sequence from strain LMG 2665. Hierarchical clustering was applied to rows and columns by Euclidean distance with no scaling, and heatmaps (*x*-axis = pPAR sRNAs, *y*-axis = sRNAs hit in other *P. ananatis* strain or bacterial species) were generated using ClustVis.

To visualize the degree of conservation of the identified pPAR sRNAs, a genome-wide analysis was performed to identify sequences similar to those of the pPAR sRNAs for several bacterial species both within and outside the genus *Pantoea* were conducted. Nearly all of the pPAR sRNAs are highly conserved within the *P. ananatis* strains, and a large portion of sRNAs was also well conserved within the genus *Pantoea* (Figure 8A). As some *P. ananatis* genomes are in draft form, it is possible that some sRNAs have not yet been assembled, accounting for low levels of conservation within the species. However, it will be interesting to determine experimentally if some sRNAs are specific within *P. ananatis* or within the genus *Pantoea* as far fewer pPAR sRNAs are conserved across different enterobacterial species (Figure 8B).

### Experimental Validation and Characterization of Individual sRNAs

**FIGURE 9:**
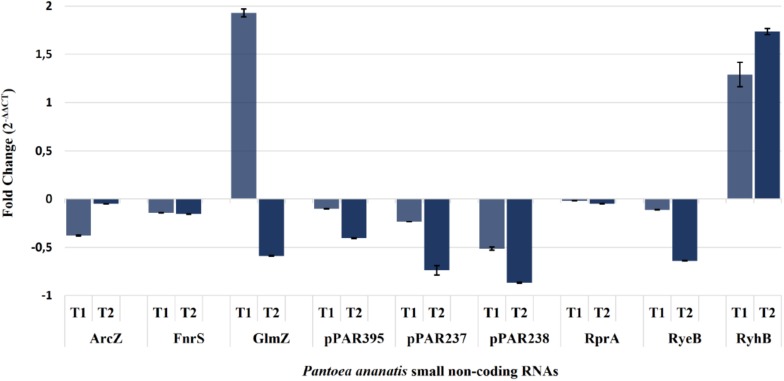
Quantification of selected *Pantoea ananatis* sRNAs (pPAR sRNAs). Transcript levels of a selected set of sRNAs in the *hfq* mutant of *P. ananatis* LMG 2665^T^ (Δ*hfq*) relative to the wild-type (WT) *P. ananatis* LMG 2665^T^ at low (T1; OD_600nm_ = 0.2) and high cell density (T2; OD_600nm_ = 0.6) were quantified using qRT-PCR. The fold change of sRNA expression in Δ*hfq* calibrated by WT expression is shown.

Expression of the *arc*Z, *fnr*S, *glm*Z, *rpr*A, *rye*B, *ryh*B2, *pPAR*237, *pPAR*238, and *pPAR*395 sRNA genes was quantified in the *P. ananatis hfq*-mutant strain relative to WT using qRT-PCR (Figure 9). The resulting expression profile of aforementioned sRNA transcript levels (except *glmZ* and *ryhB2*) was decreased in the absence of *hfq*, which was in agreement with the depth plots analysis (Figure 7). In WT *P. ananatis*, *glmZ* expression is likely repressed at low cell density (OD_600_ = 0.2) and increased at high cell density (OD_600_ = 0.6) in a Hfq-dependent manner as the opposite expression levels were observed in *hfq*-mutant *P. ananatis* where abundances of *glmZ* transcripts were detected at low cell density but not at high cell density condition. Similarly, Hfq may negatively affect *ryhB2* expression, as the abundances of *ryhB2* transcript in WT at low and high cell density conditions were both low relative to the *hfq*-mutant *ryhB2* levels. The TSS of FnrS, GlmZ, pPAR237, pPAR238, and pPAR395 was determined by 5′-RACE analysis. Their predicted structures, sequence, and targets are reported in Supplementary Figure S6 and Supplementary Table S4.

## Discussion

In the present study, we investigated the functional role of Hfq in the pathogenesis of the Gram-negative phytopathogen *P. ananatis*, and demonstrated that Hfq is important for motility, AHL and biofilm formation, and virulence of the pathogen. We also identified several putative sRNAs, which include known and novel sRNAs that are Hfq-dependent for their abundances in *P. ananatis*. The pleiotropic phenotypes caused by *hfq* mutation is due to global post-transcriptional gene regulation operated by Hfq and Hfq-dependent sRNAs that modulate stress response and virulence of numerous bacterial pathogens (Chao and Vogel, 2010). The ability of *P. ananatis* to survive in diverse ecological niches and to successfully infect susceptible plant hosts requires a timely and collective regulation of cellular functions in response to environmental conditions.

Inactivation of *hfq* in bacteria generally results in pleiotropic effects, of which growth retardation is common. Decreased growth rate has been reported in *hfq*-attenuated bacteria such as *Acinetobacter baumannii* (Kuo et al., 2017), *Haemophilus influenzae* (Hempel et al., 2013), *Yersinia enterocolitica* (Kakoschke et al., 2016), and the plant pathogens *A. tumefaciens* (Wilms et al., 2012a) and *P. carotovorum* (Wang et al., 2018). This phenotype was consistent with the *P. ananatis hfq* deletion mutant growing *in vitro*; however, this alteration did not prevent *P. ananatis* from entering logarithmic growth phase and eventually reaching the wild-type cell density at a stationary phase which was also observed *in planta* (Supplementary Figure S4). Unlike the *E. amylovora hfq* mutant (Zeng et al., 2013), which exhibited reduced growth in an immature pear fruit infection model, the *P. ananatis hfq* mutant strain was able to reach a population density comparable to that of the wild-type strain when inoculated into onion, indicating that the abolishment of virulence in the *hfq-*mutant *P. ananatis* was not due to a growth defect.

The loss of *hfq* gives rise to impairment of important virulence determinants such as motility in bacterial pathogens. Impaired motility affects the overall fitness of a bacterium as a pathogen as it disables attachment and dispersal of the pathogen in the host. This, in turn, results in the diminished invasion, colonization, and hence virulence (Sittka et al., 2007; Kulesus et al., 2008). In enterobacterial pathogens, Hfq and Hfq-dependent sRNAs control flagellar-based motility. For example, in *E. coli*, multiple sRNAs including ArcZ, OmrAB, OxyS, and RyeB/SdsR have been shown to modulate the expression and/or translation of *flhDC*, the master regulator of flagellar biosynthesis (De Lay and Gottesman, 2012). In the phytopathogen, *E. amylovora*, the sRNAs ArcZ, OmrAB, and RmaA have been found to regulate *flhDC* at both transcriptional and post-transcriptional levels (Schachterle and Sundin, 2019; Schachterle et al., 2019). In this way, the integration of different environmental cues is achieved through several sRNAs, allowing fine-tuning of flagellar expression and production.

The lack of swimming motility displayed by the *P. ananatis hfq* mutant clearly demonstrates the role of Hfq in regulating flagellar motility. In a previous study by Weller-Stuart et al. (2016), a *P. ananatis flg*K mutant deficient in flagellar assembly enzyme, FlgK, was abolished in swimming motility and pathogenicity in onion seedlings. Together with the current study, these findings suggest that flagellar motility is required for the virulence of *P. ananatis*, and this trait is regulated by functional Hfq. Similarly, in *P. carotovorum* (Wang et al., 2018) and *Serratia* sp. ATCC 39006 (Wilf et al., 2013; Hampton et al., 2016), attenuation of flagellar motility was observed in *hfq*-deletion mutant strains, as an expression of the *flhD*C genes was dependent on Hfq. Given that sRNAs namely, ArcZ, OmrAB, and RyeB/SdsR, were also identified in our sRNA sequencing data (Supplementary Table S2), and that their wild-type transcript levels are dependent on the functional copy of *hfq* (Figure 9), we hypothesize that Hfq, in conjunction with the identified sRNAs, may regulate the flagellar motility of *P. ananatis* in a similar manner as the other enterobacterial species.

In addition to impaired motility, disruption of *hfq* in Gram-negative pathogens often results in reduced biofilm formation (Kulesus et al., 2008; Monteiro et al., 2012; Zeng et al., 2013; Wang et al., 2018). One possible explanation for this phenotype is an effect on QS-mediated regulation of motility and biofilm formation. As biofilm formation is a developmental and co-operative process, the process necessitates cell to cell communication that enables perception of the signals generated from the community. The signal or information is packaged in the form of autoinducer molecules, acylated homoserine lactones (AHLs), or may be communicated to the QS circuit by secondary signaling molecule such as cyclic dimeric guanosine monophosphate (c-di-GMP) (Castiblanco and Sundin, 2016). Through protein phospho-relay, signals resulting from high cell density reach Hfq-dependent sRNAs which initiate the expression of genes required for the extracellular matrix synthesis and maturation of biofilm (Lenz et al., 2004; Kay et al., 2006; Tu and Bassler, 2007).

Consistent with the findings that in some bacteria, Hfq positively regulates biofilm production, a significantly reduced amount of biofilm was formed by the *P. ananatis* strain lacking an *hfq* gene. Interestingly, a decreased level of extracellular AHLs diffused in the supernatant of an *hfq* mutant overnight culture was detected by the CV026 biosensor, suggesting a potential role of Hfq in the positive regulation of AHL synthesis. According to Pomini et al. (2006), the main *P. ananatis*-derived AHL molecule is *N*-hexaonoyl-L-homoserine lactone (C6-HSL) and is synthesized by the LuxI homolog EanI, while LuxR homolog EanR is a transcriptional regulator that down-regulates the expression of *ean*IR operon in the absence of AHL (Morohoshi et al., 2007). Functional characterization further demonstrated that *eanI* or AHL synthase was required for the formation of biofilm, EPS, and pathogenicity of *P. ananatis* (Morohoshi et al., 2007; Sibanda et al., 2016). Thus, decreased production of AHL in the *hfq-*mutant strain of *P. ananatis* would mean down-regulation of *ean*I, virulence-related genes, as well as the genes within the QS regulon (Sibanda et al., 2018).

In the present study, two putative novel Hfq-dependent sRNAs were identified in the vicinity of the *ean*IR genes namely, pPAR237 and pPAR238 (Supplementary Figure S7A). These sRNAs are partially antisense to each other and are abundantly present in the low cell density condition but are drastically reduced in abundance at high cell density (Figure 9). In contrast to the wild-type expression levels, expression of pPAR237 and pPAR238 was almost non-existent in the two cell density conditions in the *P. ananatis hfq*-mutant strain (Figure 9). The decreased transcript levels of pPAR237 and pPAR238 in *hfq*-mutant relative to WT *P. ananatis* were validated experimentally by qRT-PCR, reinforcing the idea that the expression of these sRNAs is dependent on cell density and Hfq. Potential pairing sites of pPAR237 and pPAR238 to *ean*I and *ean*R were predicted *in silico* using IntaRNA (Supplementary Figures S7B–D). Further experimental confirmation of their interaction will indicate the role of pPAR237 and pPAR238 in QS through modulation of AHL synthesis in *P. ananatis*. Moreover, it will be also interesting to determine whether there are other upstream and downstream transcriptional or translational regulators of putative sRNAs pPAR237 and pPAR238. This is the case in *V. cholera* and *Pseudomonas aeruginosa* whose Hfq-dependent sRNA Qrr 1,2,3, and 4 and RsmY are transcriptionally activated by LuxO and GacA, respectively, and are used to repress transcription of *hapR* or sequester translational regulator RsmA (Lenz et al., 2004; Kay et al., 2006; Tu and Bassler, 2007; Brencic et al., 2009).

To date, factors that contribute to the pathogenicity of *P. ananatis* have been characterized, resulting in an expansion in our understanding of virulence mechanisms of this pathogen. A collective regulation of all virulence traits seems likely for the success and persistence of *P. ananatis* in hostile environments, and this can be achieved through Hfq and its global regulatory networks constituted by Hfq-dependent sRNAs. Overall, this study provided valuable insights into the essential role of Hfq in regulating different virulence traits of *P. ananatis*. A total of 276 sRNAs were identified that are affected in abundance by Hfq at low and high cell density conditions. These sRNAs include those that are well characterized as well as novel putative sRNAs that may possess novel function involved in the QS of *P. ananatis*.

## Data Availability

The datasets generated for this study can be found in the NCBI BioProject PRJNA550544.

## Author Contributions

GS, JS, DS, LM, TC, and GWS conceived and designed the present study. GS conducted the mutagenesis, phenotypic assays, and sRNA validation experiments. JS compiled the custom python script and performed the bioinformatics analyses of sRNA sequencing data. GS and JS wrote the manuscript in consultation with DS, LM, TC, and GWS. All authors contributed to and approved the final version of the manuscript.

## Conflict of Interest Statement

The authors declare that the research was conducted in the absence of any commercial or financial relationships that could be construed as a potential conflict of interest.

## Footnotes

1 http://rna.tbi.univie.ac.at

2 http://rna.informatik.uni-freiburg.de

## Supplementary Material

FIGURE S1 Southern blot validation of *hfq* knock-out mutation in *Pantoea ananatis*. Genomic DNA of the wild-type (WT) and *hfq* mutant (Δ*hfq*) strains of *P. ananatis* LMG 2665^T^ digested with *Eco*RI and *Hin*dIII restriction enzymes was hybridized to a DIG-labeled probe (a partial amplicon of kanamycin resistance gene). Positive detection of the antibiotic marker was observed in the Δ*hfq* strains of *P. ananatis* LMG 2665^T^ (lanes 2–8). WT of *P. ananatis* LMG 2665^T^ DNA was used as a negative control (lane 1) whereas unlabeled probe was used as a positive control (lane 9).

FIGURE S2 Colony PCR verification of *hfq* knock-out mutation in *Pantoea ananatis*. A colony PCR confirmation of insertion of kanamycin resistance gene in the *hfq* gene region using Test primers (Table 2) *hfq* mutant (Δ*hfq*) strains of *P. ananatis* LMG 2665^T^. L represents a molecular ladder and the sizes of its prominent bands 1, 3, and 6 kilo basepairs (kb) are indicated below. A wild-type (WT) colony of *P. ananatis* LMG 2665^T^ was used as a negative control (lane 1; 500 bp). Insertion of kanamycin resistance marker is shown in colony PCRs of *hfq* mutant (Δ*hfq*) strains of *P. ananatis* LMG 2665^T^ (lanes 2, 3, and 4; 1.5 kb).

FIGURE S3 *In vitro* growth assay. Growths of wild-type (WT), *hfq* mutant (Δ*hfq*), and *hfq* complementing (Δ*hfq* pBBR1MCS::*hfq*) strains of *Pantoea ananatis* LMG 2665^T^ in LB broth at 28°C. The growth was monitored for 20 h at optical density 600 nm (OD_600_) and the mean OD_600_ readings of the three replicates for each *P. ananatis* LMG 2665^T^ strains were plotted. Solid line (yellow) represents WT, dashed line (purple) Δ*hfq*, and dotted line (green) Δ*hfq* pBBR1MCS::*hfq*. Asterisks denote significance differences (*P* < 0.05) in the absorbance of Δ*hfq* relative to WT *P. ananatis* LMG 2665^T^.

FIGURE S4 *In planta* growth assay. **(A)** Disease progression in onion scales inoculated with wild-type (WT), *hfq* mutant (Δ*hfq*), and *hfq* complementing [Δ*hfq* (pBBR1MCS::*hfq*)] strains of *P. ananatis* LMG 2665^T^, and incubated for 5 days post inoculation (dpi). **(B)** *In planta* populations of WT, Δ*hfq*, and Δ*hfq* (pBBR1MCS::*hfq*) strains of *P. ananatis* LMG 2665^T^ in onion scales measured for 5 dpi. The mean CFUs of three replicates for each strain from two independent experiments were plotted. Solid line (yellow) represents WT, dashed line (purple) Δ*hfq*, and dotted line (green) Δ*hfq* (pBBR1MCS::*hfq*).

FIGURE S5 Logarithmic plot of the number of putative small RNAs (sRNAs) identified in *Pantoea ananatis* LMG 2665^T^ (pPAR sRNA) as a function of the threshold selected for calling sRNAs. This was generated by calling putative sRNAs across a range of thresholds using the custom script (see Supplementary Data Sheet S1 in the section “peak_ID.py”).

FIGURE S6 *In silico* prediction of selected *Pantoea ananatis* sRNAs (pPAR sRNA) secondary structure. Secondary structures of *P. ananatis* LMG 2665^T^ sRNAs **(A)** FnrS, **(B)** GlmZ, **(C)** pPAR 237, **(D)** pPAR 238, and **(E)** pPAR 395 were predicted based on a minimum free energy model provided by RNAfold (http://rna.tbi.univie.ac.at).

FIGURE S7 Putative interaction of pPAR237 and pPAR238 to *ean*IR in *Pantoea ananatis* LMG 2665^T^. **(A)** Location of pPAR237 and pPAR238. *In silico* predicted interaction of pPAR237 (red) to *ean*IR (black): **(B)** *ean*I upstream sequence (energy: −8.62323 kcal/mol; hybridization energy: −23.5). **(C)** *ean*R coding sequence (energy: −13.63700 kcal/mol, hybridization energy: −39.4) and **(D)** *in silico* predicted interaction of pPAR238 (red) to *ean*I (black) upstream sequence (energy: −7.83954 kcal/mol, hybridization energy: −12.0).

TABLE S1 Summary of sRNA sequencing reads obtained and filtered for use in sRNA identification.

TABLE S2 A list of sRNAs identified, their genomic coordinates, sequences, and selected characteristics.

TABLE S3 A list of sRNAs that has significant abundance difference between WT and *hfq* mutant strains of *Pantoea ananatis.*

TABLE S4 A list of predicted targets of selected sRNAs.

DATA SHEET S1 A custom phython script compiled for bioinformatic analyses of sRNA sequencing data.
