# Supplementary figures and images for: Functional Characterization of a Global Virulence Regulator Hfq and Identification of Hfq-Dependent sRNAs in the Plant Pathogen Pantoea ananatis
Source: Front Microbiol. 2019 Sep 11;10:2075. doi: 10.3389/fmicb.2019.02075 (PMC6749038; doi:10.3389/fmicb.2019.02075)

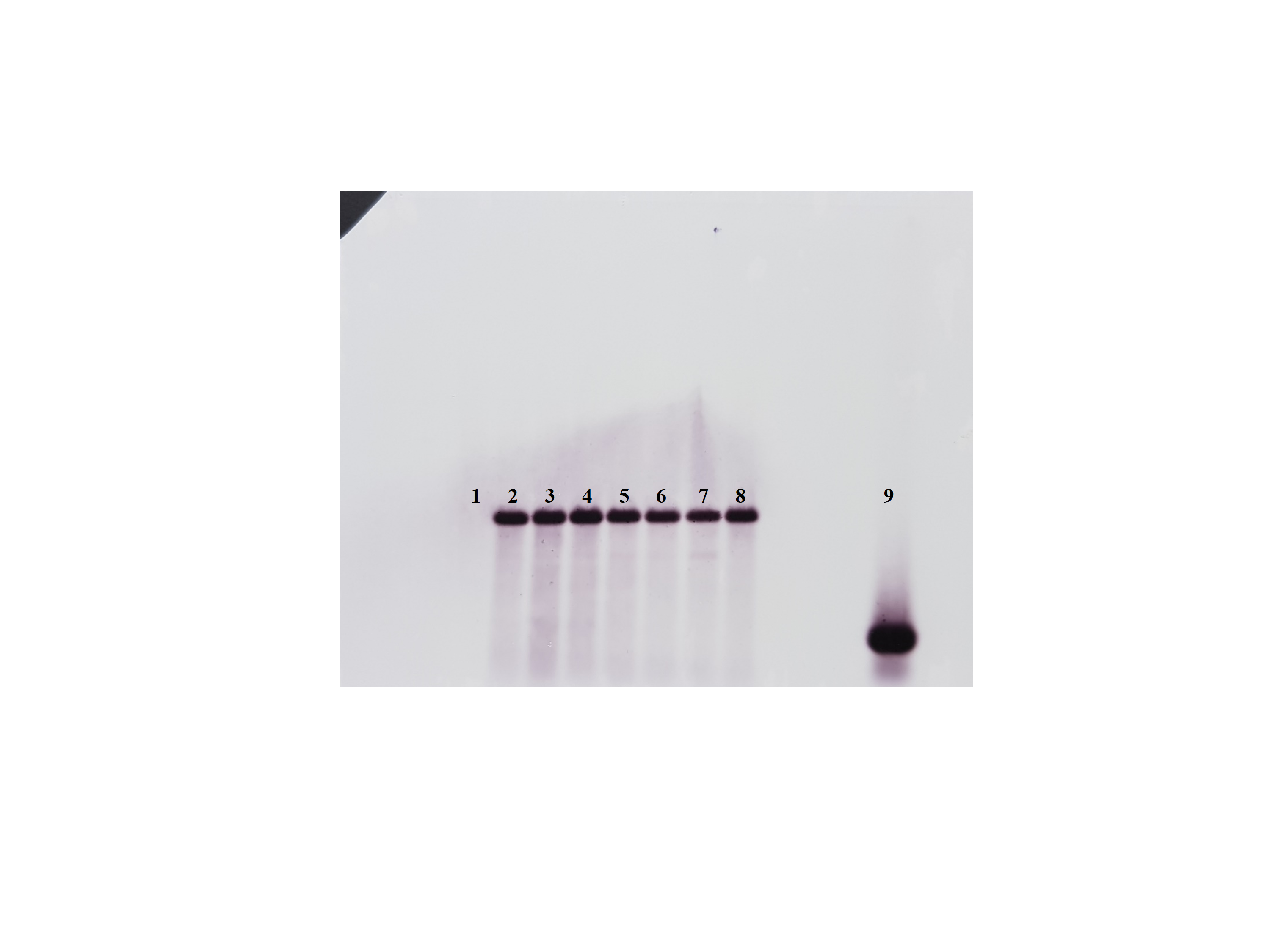

Supplement: FIGURE S1 — Southern blot validation of hfq knock-out mutation in Pantoea ananatis. Genomic DNA of the wild-type (WT) and hfq mutant (Δhfq) strains of P. ananatis LMG 2665T digested with EcoRI and HindIII restriction enzymes was hybridized to a DIG-labeled probe (a partial amplicon of kanamycin resistance gene). Positive detection of the antibiotic marker was observed in the Δhfq strains of P. ananatis LMG 2665T (lanes 2–8). WT of P. ananatis LMG 2665T DNA was used as a negative control (lane 1) whereas unlabeled probe was used as a positive control (lane 9). [file Image_1.TIF]

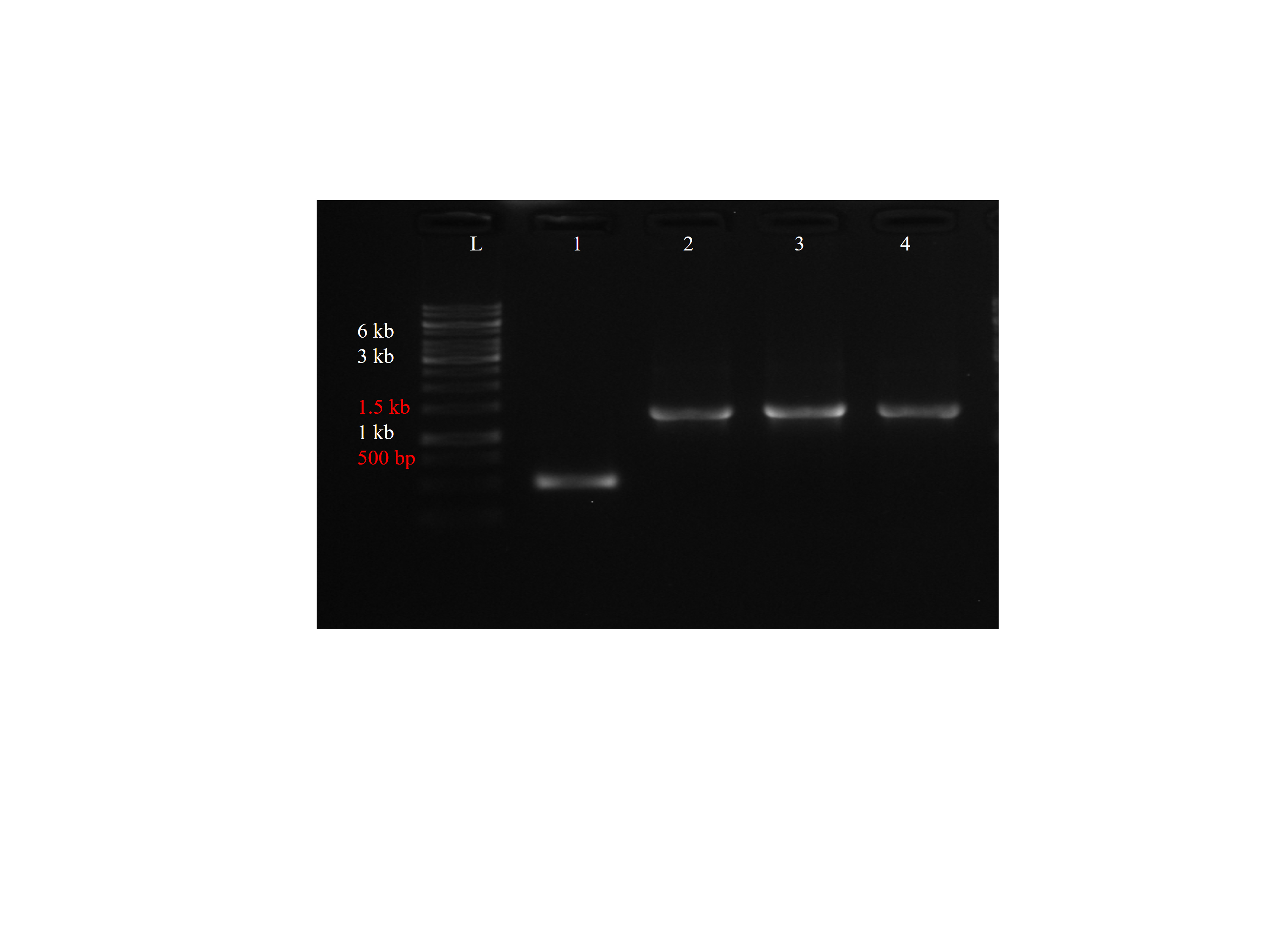

Supplement: FIGURE S2 — Colony PCR verification of hfq knock-out mutation in Pantoea ananatis. A colony PCR confirmation of insertion of kanamycin resistance gene in the hfq gene region using Test primers (Table 2) hfq mutant (Δhfq) strains of P. ananatis LMG 2665T. L represents a molecular ladder and the sizes of its prominent bands 1, 3, and 6 kilo basepairs (kb) are indicated below. A wild-type (WT) colony of P. ananatis LMG 2665T was used as a negative control (lane 1; 500 bp). Insertion of kanamycin resistance marker is shown in colony PCRs of hfq mutant (Δhfq) strains of P. ananatis LMG 2665T (lanes 2, 3, and 4; 1.5 kb). [file Image_2.TIF]

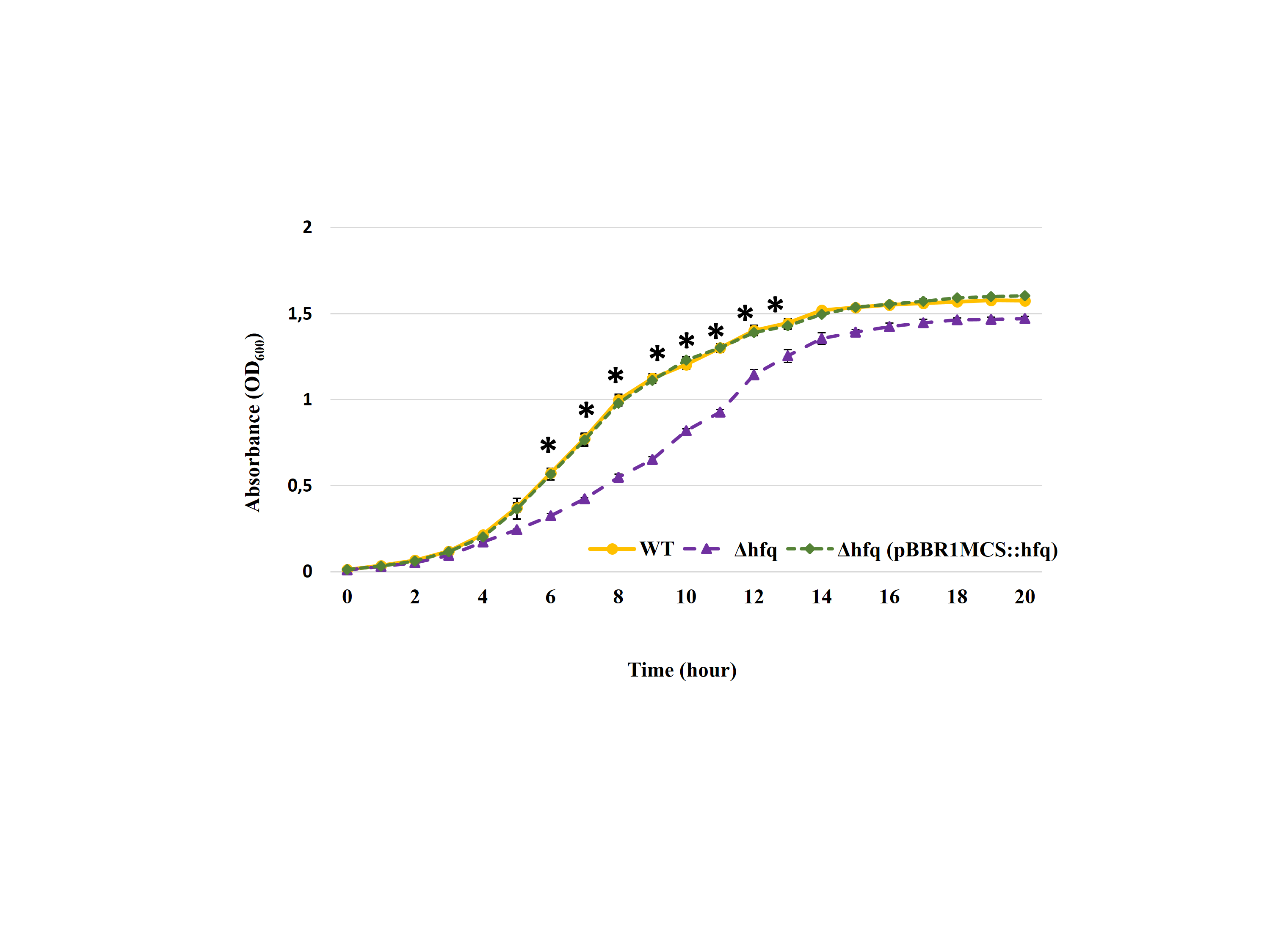

Supplement: FIGURE S3 — In vitro growth assay. Growths of wild-type (WT), hfq mutant (Δhfq), and hfq complementing (Δhfq pBBR1MCS::hfq) strains of Pantoea ananatis LMG 2665T in LB broth at 28°C. The growth was monitored for 20 h at optical density 600 nm (OD600) and the mean OD600 readings of the three replicates for each P. ananatis LMG 2665T strains were plotted. Solid line (yellow) represents WT, dashed line (purple) Δhfq, and dotted line (green) Δhfq pBBR1MCS::hfq. Asterisks denote significance differences (P < 0.05) in the absorbance of Δhfq relative to WT P. ananatis LMG 2665T. [file Image_3.TIF]

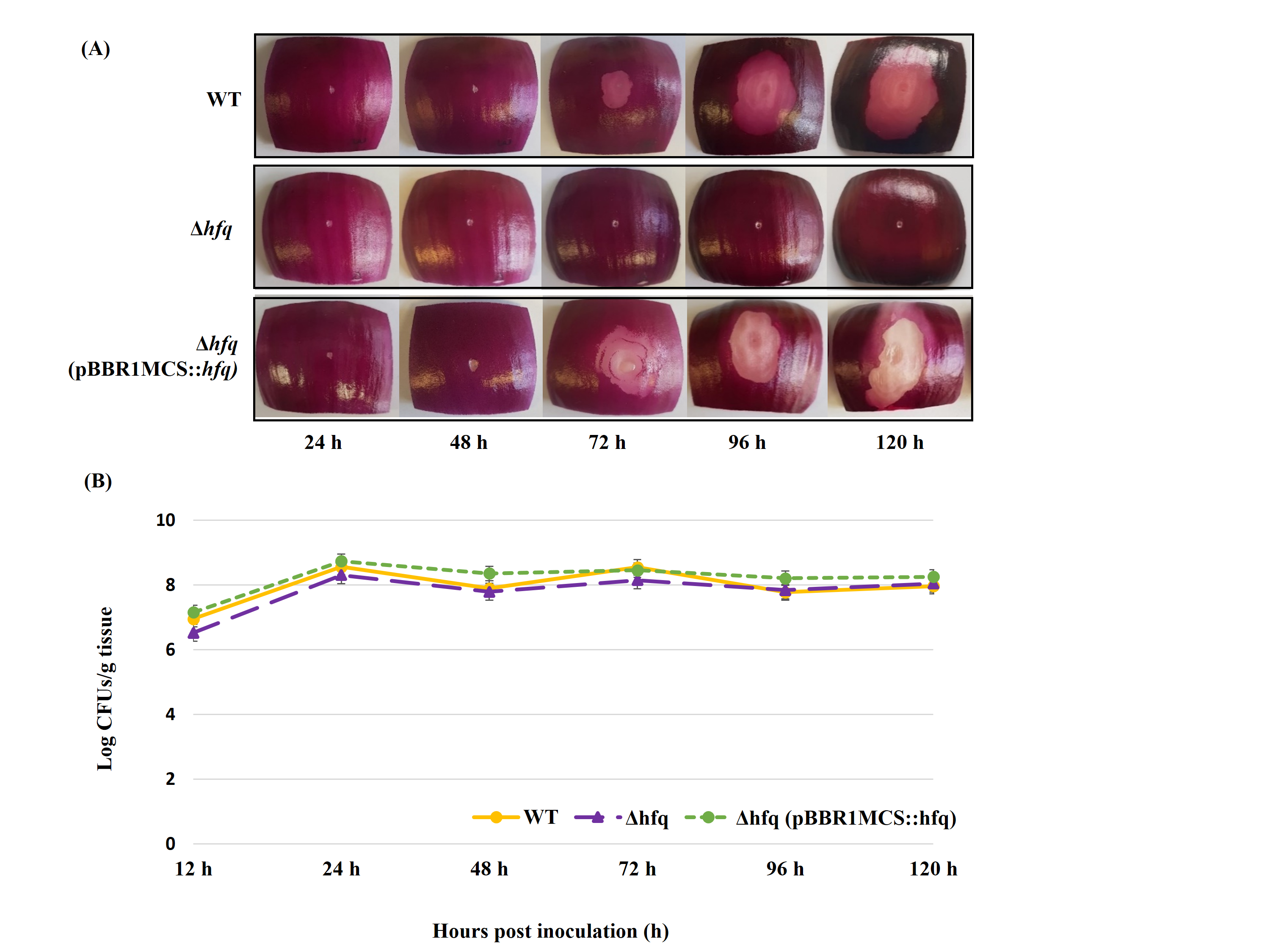

Supplement: FIGURE S4 — In planta growth assay. (A) Disease progression in onion scales inoculated with wild-type (WT), hfq mutant (Δhfq), and hfq complementing [Δhfq (pBBR1MCS::hfq)] strains of P. ananatis LMG 2665T, and incubated for 5 days post inoculation (dpi). (B) In planta populations of WT, Δhfq, and Δhfq (pBBR1MCS::hfq) strains of P. ananatis LMG 2665T in onion scales measured for 5 dpi. The mean CFUs of three replicates for each strain from two independent experiments were plotted. Solid line (yellow) represents WT, dashed line (purple) Δhfq, and dotted line (green) Δhfq (pBBR1MCS::hfq). [file Image_4.TIF]

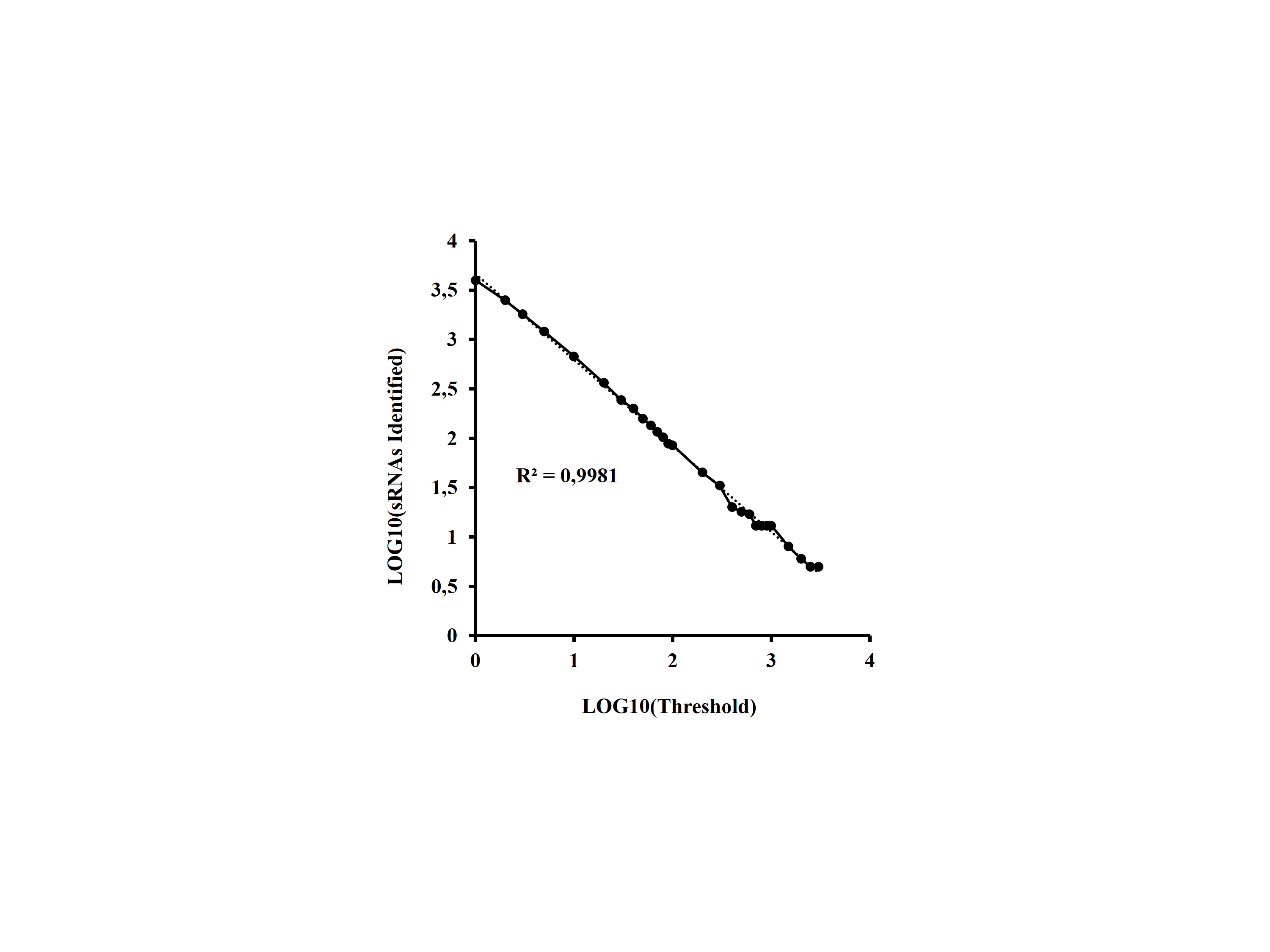

Supplement: FIGURE S5 — Logarithmic plot of the number of putative small RNAs (sRNAs) identified in Pantoea ananatis LMG 2665T (pPAR sRNA) as a function of the threshold selected for calling sRNAs. This was generated by calling putative sRNAs across a range of thresholds using the custom script (see Supplementary Data Sheet S1 in the section “peak_ID.py”). [file Image_5.TIF]

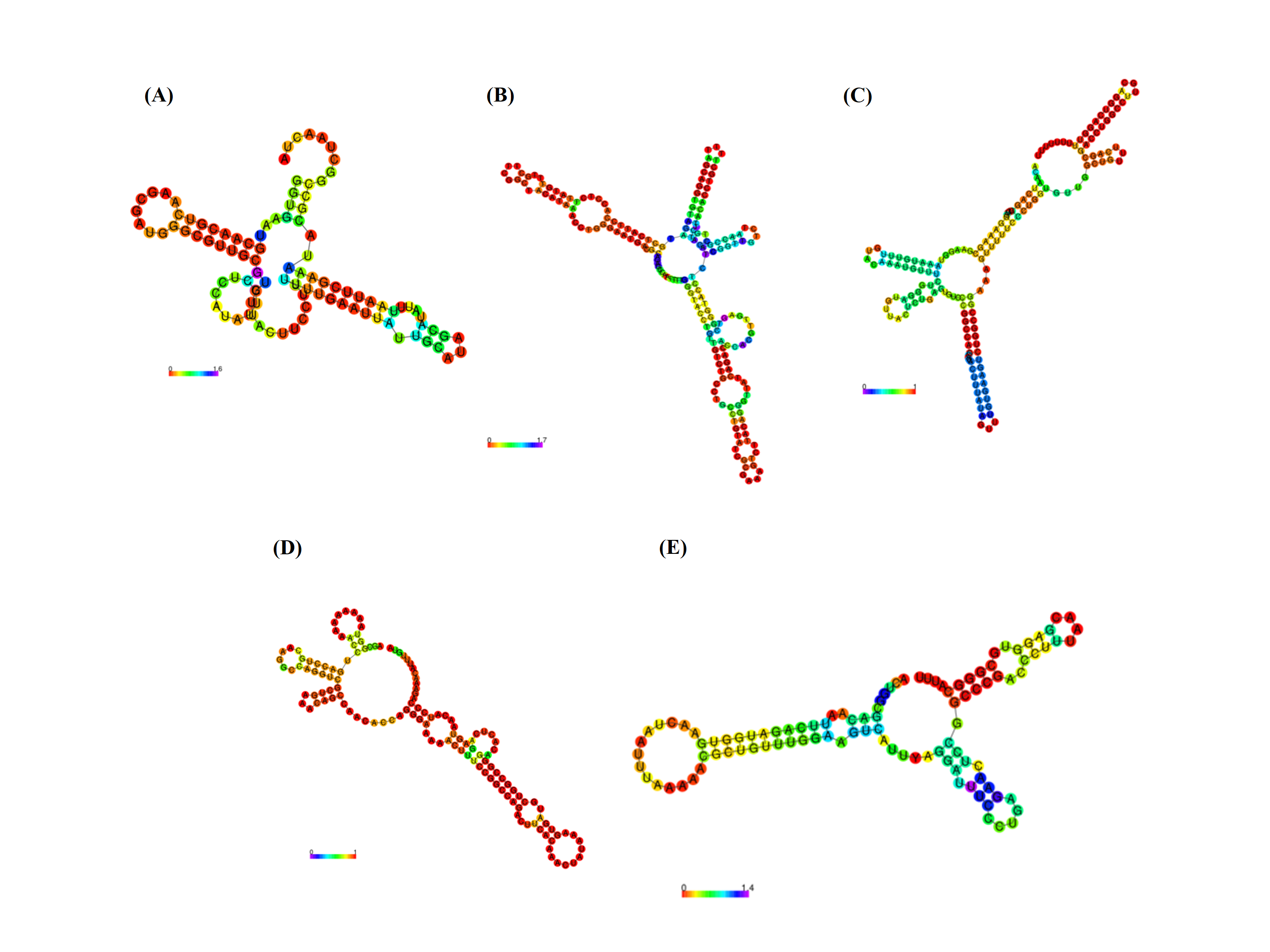

Supplement: FIGURE S6 — In silico prediction of selected Pantoea ananatis sRNAs (pPAR sRNA) secondary structure. Secondary structures of P. ananatis LMG 2665T sRNAs (A) FnrS, (B) GlmZ, (C) pPAR 237, (D) pPAR 238, and (E) pPAR 395 were predicted based on a minimum free energy model provided by RNAfold (http://rna.tbi.univie.ac.at). [file Image_6.TIF]

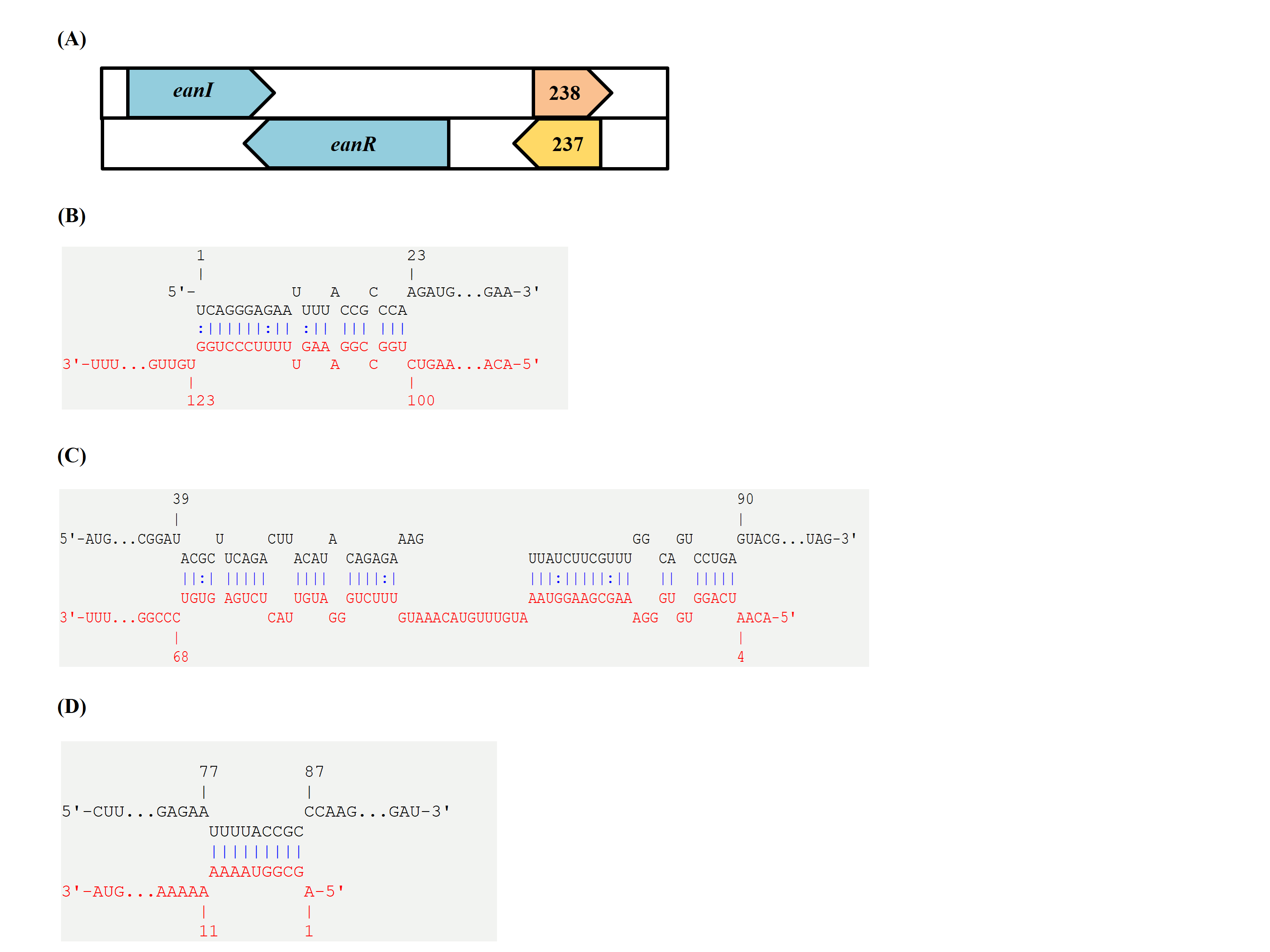

Supplement: FIGURE S7 — Putative interaction of pPAR237 and pPAR238 to eanIR in Pantoea ananatis LMG 2665T. (A) Location of pPAR237 and pPAR238. In silico predicted interaction of pPAR237 (red) to eanIR (black): (B) eanI upstream sequence (energy: −8.62323 kcal/mol; hybridization energy: −23.5). (C) eanR coding sequence (energy: −13.63700 kcal/mol, hybridization energy: −39.4) and (D) in silico predicted interaction of pPAR238 (red) to eanI (black) upstream sequence (energy: −7.83954 kcal/mol, hybridization energy: −12.0). [file Image_7.TIF]
