# Supplementary material for: Functional Characterization of a Global Virulence Regulator Hfq and Identification of Hfq-Dependent sRNAs in the Plant Pathogen Pantoea ananatis
Source: Front Microbiol. 2019 Sep 11;10:2075. doi: 10.3389/fmicb.2019.02075 (PMC6749038; doi:10.3389/fmicb.2019.02075)
Supplement: TABLE S1 — Summary of sRNA sequencing reads obtained and filtered for use in sRNA identification. [file Table_1.DOCX]

| **S1 Table**: Summary of sRNA sequencing reads obtained and filtered for use in sRNA identification | | | | | | | | |
| --- | --- | --- | --- | --- | --- | --- | --- | --- |
| **Time Point** | **Strain** | **Rep** | **Reads Sequenced** | **Reads after trimming (%)^a^** | **Reads mapping (%)^b^** | **CDS/rRNA/tRNA removed reads** | **Intergenic/Antisense reads for sRNA identification (%)^c^** | **Accession number^d^** |
| 1 | *∆hfq* | 1 | 12805712 | 5941160 (46.4) | 5071660 (85.4) | 4474767 | 772585 (15.2) | SRX6360880 |
| 1 | *∆hfq* | 2 | 15826947 | 7669898 (48.5) | 6547118 (85.4) | 6005699 | 759382 (11.6) | SRX6360881 |
| 1 | *∆hfq* | 3 | 16510146 | 6052015 (36.7) | 5186049 (85.7) | 4416923 | 881587 (17.0) | SRX6360882 |
| 1 | wt | 1 | 14649162 | 6705591 (45.8) | 5514211 (82.2) | 4998767 | 851927 (15.4) | SRX6360883 |
| 1 | wt | 2 | 13581459 | 4764989 (35.1) | 3960973 (83.1) | 3265001 | 787185 (19.9) | SRX6360884 |
| 1 | wt | 3 | 11822001 | 3622119 (30.6) | 3054719 (84.3) | 2503109 | 610814 (20.0) | SRX6360885 |
| 2 | *∆hfq* | 1 | 17472236 | 4951444 (28.3) | 3972637 (80.2) | 3101846 | 926505 (23.3) | SRX6360886 |
| 2 | *∆hfq* | 2 | 14827958 | 5872626 (39.6) | 4789010 (81.5) | 4064192 | 869664 (18.2) | SRX6360887 |
| 2 | *∆hfq* | 3 | 13979121 | 7352684 (52.6) | 6036552 (82.1) | 5415534 | 910203 (15.1) | SRX6360878 |
| 2 | wt | 1 | 13305844 | 4869024 (36.6) | 3962410 (81.4) | 3372805 | 762242 (19.2) | SRX6360879 |
| 2 | wt | 2 | 13869254 | 3846989 (27.7) | 3182489 (82.7) | 2442902 | 794122 (25.0) | SRX6360876 |
| 2 | wt | 3 | 13381665 | 5088389 (38.0) | 4250962 (83.5) | 3567480 | 794023 (18.7) | SRX6360877 |
| ^a^Percent of initial reads sequenced that passed quality filtering and adapter trimming | | | | | | | | |
| ^b^Percent of reads mapped out of reads remaining after trimming | | | | | | | | |
| ^c^Percent of mapped reads that did not map to known coding sequences, rRNAs, or tRNAs | | | | | | | | |

^d^NCBI accession number of raw RNA sequencing data
