# Supplementary material for: Functional Characterization of a Global Virulence Regulator Hfq and Identification of Hfq-Dependent sRNAs in the Plant Pathogen Pantoea ananatis
Source: Front Microbiol. 2019 Sep 11;10:2075. doi: 10.3389/fmicb.2019.02075 (PMC6749038; doi:10.3389/fmicb.2019.02075)
Supplement: DATA SHEET S1 — A custom phython script compiled for bioinformatic analyses of sRNA sequencing data. [file Data_sheet_1.PDF]

genic_filter.py

```
###############################################################################

##SAM file genic filter

#import threading
#import Queue
import sys

#Parse and store CDS sequences from annotation file
annotfile='ref/GCA_000025405.2_ASM2540v1_genomic.gff'
aFile=open(annotfile,'r')

outfile_name="sfilt_"+sys.argv[1]
outfile=open(outfile_name,'w')
plus_CDS={}
minus_CDS={}
for liner in aFile:
	liner=liner.rstrip()
	if liner.startswith("#"):
		skip=True
		#skip comments/headers
	else:
		splat=liner.split("\t")
		#print splat
		if splat[2]=="CDS" or splat[2]=="tRNA" or splat[2]=='rRNA':
			if splat[6]=="+":
				#Add to + strand list of features				>>>>> Remove
promoters and terminators too.<<<<<<<<<
				for n in range(int(splat[3])-120,int(splat[4])+120):
					plus_CDS[n]='+'
			elif splat[6]=="-":
				#Add to - strand list of features
				for n in range(int(splat[3])-120,int(splat[4])+120):
					minus_CDS[n]='-'

samfile=sys.argv[1]
#Parse sam file:
file=open(samfile,'r')
s_plus_CDS=set(plus_CDS.keys())
s_minus_CDS=set(minus_CDS.keys())
total_sam_lines=0
kept=0
removed=0
unmapped=0
for line in file:
	line=line.rstrip()
	if line.startswith("@"):
```

```
                skip=True

                outfile.write("%s\n"%line)
                #re-print sam headers
        else:
                total_sam_lines+=1
                #line=line.rstrip()
                cutup=line.split()
                location=int(cutup[3])
                if cutup[1]=='4':
                        #skip all unmapped reads
                        unmapped+=1
                elif cutup[1]=='16':

                        if location in s_minus_CDS:
                                removed+=1
                        else:
                                outfile.write("%s\n"%line)
                                kept+=1
                elif cutup[1]=='0':

                        if location in s_plus_CDS:
                                removed+=1
                        else:
                                #print line
                                kept+=1
                                outfile.write("%s\n"%line)
outfile.close()
print("%s reads in sam file"%total_sam_lines)
print("%s were unmapped"%unmapped)
print("%s mapped to CDS regions and were removed"%removed)
print("%s mapped to intergenic regions or antisense regions and were kept"%kept)



######################################################################
```

peak_ID.py

```
################################################################################

'''
read sam file
	go through genomic positions
	compute depth > one set for + strand, another for - strand

Analyze depth > one strand at a time
	estimate background using a sliding window
	ID peaks that are at least 50 bases long  X-fold above background across entire length >> test different levels

	output peak start and end, depth, background



'''
samfile=""
sam_file=open(samfile,'r')


#initialize Dictionaries



bases=0
plus_depth={}
minus_depth={}
GenomeLength=4703372
for i in range(1,GenomeLength+1):
	plus_depth[i]=0
	minus_depth[i]=0
for line in sam_file:
	line=line.rstrip()
	if line.startswith("@"):
		ignore=True
	else:
		columns=line.split()
		bases=bases+len(columns[9])
		if columns[1]=='4':
			unmapped=True
		elif columns[1]=='0':
			#plus_strand
			for x in range(int(columns[3]),int(columns[3])+len(columns[9])):
				if x<=GenomeLength:
					plus_depth[x]+=1
				else:
```

```
					plus_depth[x-GenomeLength]+=1
		elif columns[1]=='16':
			#minus_strand
			for x in range(int(columns[3]),int(columns[3])+len(columns[9])):
				if x<=GenomeLength:
					minus_depth[x]+=1
				else:
					minus_depth[x-GenomeLength]+=1

#print bases
#print bases/GenomeLength
Gbackground=float(bases)/GenomeLength
windowWidth=1000
stepSize=1
ratio_Threshold=10
pos=1
#Peak finding
fpeakLength=0
rpeakLength=0
#set initial Forward background
#fsum=0
#for x in range(1,1000):
#	fsum+=plus_depth[x]
#fbackground=fsum/1000

#set initial minus background
#rsum=0
#for x in range(1,1000):
#	rsum+=minus_depth[x]
#rbackground=rsum/1000

#outfile=open("zzz_F_test_N.txt",'w')
#Print Headers:
#for file in samfile_list:
#	outfile.write("%s\t"%file)
#outfile.write("\n")

while pos<GenomeLength:
	#fbackground=fbackground-(plus_depth[pos-1]/1000)+(plus_depth[pos+1000]/1000)
	#rbackground=rbackground-(minus_depth[pos-1]/1000)+(minus_depth[pos+1000]/1000)
	if (plus_depth[pos]/Gbackground)>ratio_Threshold:
		fpeakLength+=1
	else:
		if fpeakLength>=10:# and fpeakLength<50:
			print "+\t%s\t%s\t%s\t%s"%(pos-fpeakLength,pos-1,plus_depth[pos-
fpeakLength],plus_depth[pos-1])
		fpeakLength=0
	if (minus_depth[pos]/Gbackground)>ratio_Threshold:
```

```
                rpeakLength+=1
        else:
                if rpeakLength>=10:# and rpeakLength<50:
                        print "-\t%s\t%s\t%s\t%s"%(pos-rpeakLength,pos-1,minus_depth[pos-
rpeakLength],minus_depth[pos-1])
                rpeakLength=0
        #outfile.write("%s\t"%(pos))
        #index=0
        #for dict in f_depth_files:
        #       outfile.write("%s\t"%(dict[pos]/(float(library_size[index])/GenomeLength)))
        #       index+=1
        #outfile.write("\n")
        pos+=1
#outfile.close()


############################################################################
```

mergeList.py

```
#########################################################################

#Merge lists of putative sRNAs

infile1=open("list1.txt",'r')
infile2=open("list2.txt",'r')


time1=[]
time2=[]
for line1 in infile1:
	line1=line1.rstrip()
	time1.append(line1)
for line2 in infile2:
	line2=line2.rstrip()
	time2.append(line2)
count=0
newList=[]
for entry1 in time1:
	entry1Limits=(int(entry1.split("\t")[2]),int(entry1.split("\t")[3]))
	for entry2 in time2:
		entry2Limits=(int(entry2.split("\t")[2]),int(entry2.split("\t")[3]))

		if entry1.split("\t")[1]==entry2.split("\t")[1]:

			if entry1Limits[0] in range(entry2Limits[0],entry2Limits[1]) or entry1Limits[1] in
range(entry2Limits[0],entry2Limits[1]):
				#print entry1+"\n"+entry2+"\n\n"

	newList.append("1,2\t%s\t%s\t%s"%(entry1.split("\t")[1],min(entry1Limits[0],entry1Limits[1],en
try2Limits[0],entry2Limits[1]), max(entry1Limits[0],entry1Limits[1],entry2Limits[0],entry2Limits[1])))
				count+=1

count1=0
newNewList=[]
for entry1 in time1:
	entry1Limits=(int(entry1.split("\t")[2]),int(entry1.split("\t")[3]))
	found=False
	for entryNew in newList:
		entryNewLimits=(int(entryNew.split("\t")[2]),int(entryNew.split("\t")[3]))

		if entry1.split("\t")[1]==entryNew.split("\t")[1]:
			if entry1Limits[0] in range(entryNewLimits[0],entryNewLimits[1]) or
entry1Limits[1] in range(entryNewLimits[0],entryNewLimits[1]):
				found=True

	if found:
```

```
				skip=True
				#already in newList
		else:
				newNewList.append(entry1)
				count1+=1

count2=0
for entry2 in time2:
		entry2Limits=(int(entry2.split("\t")[2]),int(entry2.split("\t")[3]))
		found=False
		for entryNew in newList:
				entryNewLimits=(int(entryNew.split("\t")[2]),int(entryNew.split("\t")[3]))

				if entry2.split("\t")[1]==entryNew.split("\t")[1]:
						if entry2Limits[0] in range(entryNewLimits[0],entryNewLimits[1]) or
entry2Limits[1] in range(entryNewLimits[0],entryNewLimits[1]):
								found=True

		if found:
				skip=True
				#already in newList
		else:
				newNewList.append(entry2)
				count2+=1
for new in newList:
		print new

print "\n\n\n"

for newnew in newNewList:
		print newnew

print "%s found in both timepoints"%count
print "%s found in timepoint 1 only"%count1
print "%s found in timepoint 2 only"%count2

##########################################################################
```

RI_term.py
```
###########################################################################


def char_count(string, character):
	count=0
	for char in string:
		if char in character:
			count+=1

	return count


genome=open("../gina/genomes/GCA_000025405.2_ASM2540v1_genomic.fa",'r')


genome_seq=''
for line in genome:
	line=line.rstrip()
	if line.startswith(">"):
		id=line.strip(">")
	else:
		genome_seq=genome_seq+line

i=0

#list of poly-T
polyT=[]
while i<len(genome_seq)-8:
	if "TTTTTT" in genome_seq[i:i+8]:#,"T")>=6:
		polyT.append((i,i+8))
	i+=1
newPolyT=[]
previousT=(0,0)
for Ti in polyT:
	if Ti[0] in range(previousT[0],previousT[1]):
		skip=True
	else:
		newPolyT.append(Ti)
	previousT=Ti

newNewPolyT=[]
for entry in newPolyT:
	#print entry
	if char_count(genome_seq[entry[0]-4:entry[0]+2],"GC")>3:
		newNewPolyT.append(entry)
#print len(newPolyT)
```

```
newNewNewPolyT=[]
for candidate in newNewPolyT:
	if char_count(genome_seq[candidate[0]-23:candidate[0]+2],"GC")>12:
		newNewNewPolyT.append(candidate)
print "%s TTTTTTs found on DNA strand"%len(newPolyT)
print "of these, %s had 4 GCs in the last 6 bases before the poly-T"%len(newNewPolyT)
print "of these, %s had more than 50 percent GC in the last 25 bases before the poly-
T"%len(newNewNewPolyT)


annotation_file=open("../gina/genomes/GCA_000025405.2_ASM2540v1_genomic.gff",'r')
gene_ends=[]
for line in annotation_file:
	line=line.rstrip()
	if line.startswith("#"):
		ignore=True #comment
	else:
		columns=line.split("\t")
		if columns[2]=="CDS" or columns[2]=="rRNA" or columns[2]=="tRNA":
			if columns[6]=="+":
				gene_ends.append(int(columns[4]))
sRNA_file=open("NmergeList.txt",'r')
sRNA_ends=[]
for line in sRNA_file:
	line=line.rstrip()
	columns=line.split("\t")
	if columns[1]=="+":
		sRNA_ends.append(int(columns[3]))


gene_associated=[]
for cand in newNewNewPolyT:
	for end in gene_ends:
		if (cand[1]-6)-end > 0 and (cand[1]-6)-end < 120:
			gene_associated.append(cand)

sRNA_associated=[]
for cand2 in newNewNewPolyT:
	for endS in sRNA_ends:
		if (cand2[1]-6)-endS >0 and (cand2[1]-6)-endS < 80:
			sRNA_associated.append(cand2)
			print cand2
print "%s gene_associated putative Rho-independent terminator sites"%len(gene_associated)
print "%s sRNA_associated putative Rho-independent terminator sites"%len(sRNA_associated)


#########################################################################
```
